# Photophysical and Antibacterial Properties of Porphyrins Encapsulated inside Acetylated Lignin Nanoparticles

**DOI:** 10.3390/antibiotics10050513

**Published:** 2021-04-30

**Authors:** Nidia Maldonado-Carmona, Tan-Sothea Ouk, Nicolas Villandier, Claude Alain Calliste, Mário J. F. Calvete, Mariette M. Pereira, Stéphanie Leroy-Lhez

**Affiliations:** 1 PEIRENE Laboratory, Faculty of Sciences and Techniques, University of Limoges, 87060 Limoges, France; nidia.maldonado@etu.unilim.fr (N.M.-C.); tan-sothea.ouk@unilim.fr (T.-S.O.); nicolas.villandier@unilim.fr (N.V.); 2 Coimbra Chemistry Center, Department of Chemistry, University of Coimbra, 3004-535 Coimbra, Portugal; mcalvete@qui.uc.pt (M.J.F.C.); mmpereira@qui.uc.pt (M.M.P.); 3 PEIRENE Laboratory, Faculty of Pharmacy, University of Limoges, 87025 Limoges, France; claude.calliste@unilim.fr

**Keywords:** tetrapyrrolic compounds, photodynamic therapy, lignin valorization

## Abstract

Lignin has recently attracted the attention of the scientific community, as a suitable raw material for biomedical applications. In this work, acetylated lignin was used to encapsulate five different porphyrins, aiming to preserve their photophysical properties, and for further use as antibacterial treatment. The obtained nanoparticles were physically characterized, through dynamic light scattering size measurement, polydispersity index and zeta potential values. Additionally, the photophysical properties of the nanoparticles, namely UV-vis absorption, fluorescence emission, singlet oxygen production and photobleaching, were compared with those of the free porphyrins. It was found that all the porphyrins were susceptible to encapsulation, with an observed decrease in their fluorescence quantum yield and singlet oxygen production. These nanoparticles were able to exert an effective photodynamic bactericide effect (blue-LED light, 450–460 nm, 15 J/cm^2^) on *Staphylococcus aureus* and *Escherichia coli*. Furthermore, it was achieved a photodynamic bactericidal activity on an encapsulated lipophillic porphyrin, where the free porphyrin failed to diminish the bacterial survival. In this work it was demonstrated that acetylated lignin encapsulation works as a universal, cheap and green material for the delivery of porphyrins, while preserving their photophysical properties.

## 1. Introduction

Antimicrobial resistance (AMR) is one of major threats to our current way of life, with enduring humanitarian and economic consequences if not addressed assertively [1]. For instance, concerns on the negative influence of the concurrent COVID-19 pandemic over AMR, are arising [2,3], particularly from the extensive use and misuse of mechanical ventilation [4,5]. Moreover, conventional antibiotics’ bacterial resistance advocates exploring less resistance-prone therapeutic approaches [6], where photodynamic antimicrobial chemotherapy (PACT) [7] has proved as an efficient alternative in the inactivation of several bacterial strains [8,9,10,11,12,13]. This therapeutic strategy involves the concomitant use of a light source in appropriate dosage, a photosensitizer molecule and molecular oxygen, generating reactive oxygen species (ROS), namely singlet oxygen (^1^O_2_), super oxide anion (O_2_^•−^), and hydroxyl radical (HO^•^). These ROS produce oxidative stress in bacteria through a non-specific molecular target mechanism, which ensures cellular death without risk of antimicrobial resistance [14].

Concurrently, organic matrices (e.g., cellulose, chitosan, cyclodextrin) have been used for the formulation of photosensitizers, aiming for the valorization of biopolymers available in the nature. Lignin is a natural aromatic polymer, usually obtained as a paper industry by-product, containing p-coumaryl alcohol, coniferyl alcohol, and sinapyl alcohol units, in proportions that vary depending on the botanic origin of the sample [15]. Its aromatic character, easy chemical tuning and low cost of the raw material have paved the way for a current tendency of lignin valorization in biomedical, chemical and environmental applications. Specially, the role of lignin in the preparation of nanomaterials for loading and release of active substances is under growing scientific scrutiny [16].

Justifiably, the use of pristine lignin as a photosensitizer’s vehicle is hampered by its known antioxidant activity [17,18]. Consequently, it is not surprising the lack of examples in the literature of lignin as part of a photodynamic treatment [19]. Our latest work reported the use of acetylated lignin nanoparticles as a vehicle for 5,10,15,20-tetrakis (4-hydroxyphenyl)-21H,23H-porphine (THPP), and its efficient antibacterial effect against Gram-positive bacteria [20]. Here, we concluded that the use of acetylated lignin as vehicle might be highly advantageous, for weak and non-hydrophillic photosensitizers. Additionally, improvements were foreseen that would enable the killing of Gram-negative bacteria, which were unaffected by the encapsulated THPP.

Herein, we report the encapsulation inside acetylated lignin nanoparticles of THPP and four derived porphyrins, 5,10,15,20-tetrakis (4-acetyloxyphenyl)-21H,23H-porphine, T(OAc)PP; zinc (II) 5,10,15,20-tetrakis (4-hydroxyphenyl)-21H,23H-porphine, ZnTHPP; 5,10,15,20-tetrakis(4-(3-*N*,*N*,*N*-trimethylammoniumpropoxy)-phenyl)-21H,23H-porphine bromine, T(MAP)PP and 5,10,15,20-tetrakis (4-(3-hydroxy)propyloxypyridyl)-21H,23H-porphine bromine, T(PrOH)PyP. The nanoparticles were characterized through dynamic light scattering (DLS), zeta potential, UV-vis absorption, fluorescence, and singlet oxygen production through electron paramagnetic resonance (EPR). The nanoparticles were tested against *Staphylococcus aureus* and *Escherichia coli*, under blue-LED light irradiation (450–460 nm, 15 J/cm^2^).

## 2. Results and Discussion

### 2.1. Porphyrins Synthesis

**Scheme 1:**
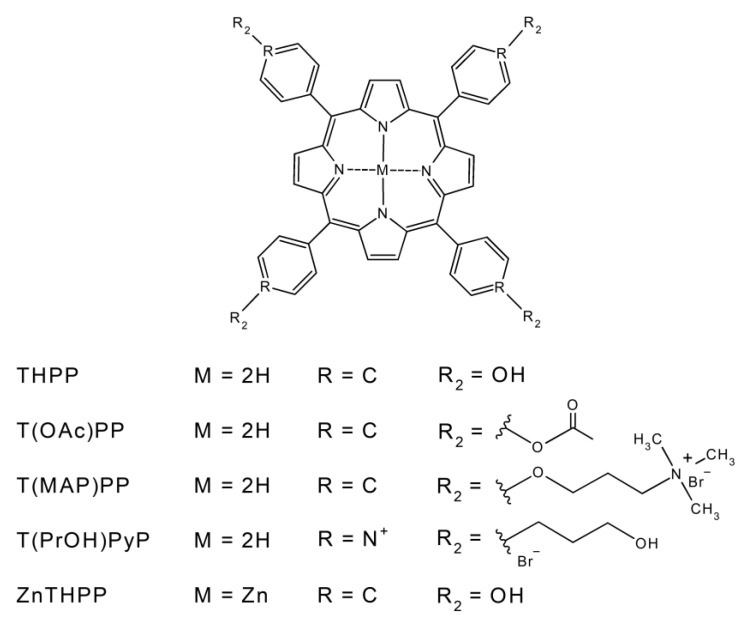
Structure of the porphyrins used along the present work.

Different porphyrins were synthesized, accordingly to already published methods, as shown in Scheme 1. These porphyrins were intended to be structural derivatives of 5,10,15,20-tetrakis (4-hydroxyphenyl)-21H,23H-porphine (THPP), by blocking the hydroxyl moiety with an acetyl group (5,10,15,20-tetrakis (4-acetyloxyphenyl)-21H,23H-porphine, T(OAc)PP) [21,22], through its metallation with a divalent metal (zinc (II) 5,10,15,20-tetrakis (4-hydroxyphenyl)-21H,23H-porphine, ZnTHPP) [23,24,25], and through the formation of a cationic porphyrin (5,10,15,20-tetrakis(4-(3-*N*,*N*,*N*-trimethylammoniumpropoxy)-phenyl)-21H,23H-porphine bromine, T(MAP)PP) [12,26]. Additionally, a cationic porphyrin with four free hydroxyl moieties was synthesized (5,10,15,20-tetrakis (4-(3-hydroxy)propyloxypyridyl)-21H,23H-porphine bromine, T(PrOH)PyP) [12], allowing a comparison between two cationic porphyrins with or without free hydroxyl moieties. The role of hydroxyl seems to be important for their hydrophilic character, as solubility and encapsulation efficiency, as well as for their possible antioxidant properties [27]. These targeted compounds have been designed for the sake of comparison and for a better understanding of the hydroxyl group role in the whole studied system.

### 2.2. Preparation of Acetylated Lignin Nanoparticles

Porphyrin-loaded acetylated lignin nanoparticles were prepared as previously reported [20], and as described in detail in Materials and Methods 4.3. Nanoparticles were obtained and kept as suspensions in distilled water, during the whole characterization and evaluation process.

### 2.3. Physical Properties of Acetylated Lignin Nanoparticles

The obtained nanoparticles suspensions were physically characterized. The size distribution was analyzed through dynamic light scattering (DLS), which provides the hydrodynamic size of any particle. Usually, the hydrodynamic size takes into account the ions that may surround a particle, so can differ from the size obtained through other methods, such as microscopic observations. Previous research has demonstrated that the hydrodynamic size correlates with the size measured through transmission electron microscopy (TEM) observations, for acetylated lignin nanoparticles [20]. The obtained size, the polydispersity index (PDI) and the zeta potential (Table 1) provide information of the stability and the way that nanoparticles interact.

**Figure 1:**
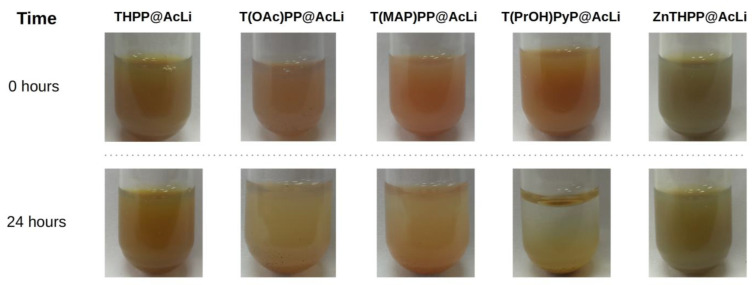
Sedimentation of the acetylated lignin nanoparticles, as a function of time.

The obtained values were compared with the empty acetylated lignin nanoparticles (@AcLi). We could observe that the presence of the porphyrins affects the size of the obtained nanoparticles, this being more evident for the cationic porphyrins, which have a size of around 1 µm, with a wide D_95_ distribution. The resulting size of the cationic porphyrins nanoparticles could be due to the rejection between the cationic charges, preventing the formation of tight nanoparticles. Furthermore, the encapsulation of cationic porphyrins affected the PDI and the zeta potential. Normally, suspensions with a PDI below 0.2 and zeta potentials above ±20 mV are desirable, which ensure that the suspension preserves its efficiency and its proper dosage. Both nanoparticles obtained with the cationic porphyrins have PDI above 0.2 and zeta potentials close to zero, resulting in suspensions that quickly sediment over time (Figure 1), with T(PrOH)PyP@AcLi being the quickest to sediment, as these nanoparticles are also the largest, with a size difference of 462 nm with respect to T(MAP)PP@AcLi nanoparticles. The difference of size seems to overweight the observed values of PDI and zeta potential, which indicated that T(MAP)PP@AcLi would tend to sediment faster than T(PrOH)PyP@AcLi [28]. In all cases, a gentle shake was enough to suspend the nanoparticles again.

### 2.4. Photophysical Properties

Previous studies [20] have demonstrated that THPP encapsulated inside acetylated lignin nanoparticles conserved their main photophysical features (i.e., absorption and emission spectra), as well as their capacity to generate singlet oxygen under light irradiation. In order to investigate the impact of the metallation, the blockage of the hydroxyl moieties, and the presence of positive charges in the porphyrins, the nanoparticles library, as well as the free porphyrins, were characterized on these different parameters.

#### 2.4.1. UV-Vis Absorption Characterization

Nanoparticles absorption spectra was measured on phosphate buffer 0.01 M pH 7 and compared with the absorption spectra of the single porphyrins in DMF or in PB pH 7 (Table 2). The obtained raw spectra for the nanoparticles were blanked from the light dispersion effect of nanoparticles. Similar treatment was used for all UV-vis spectra presented in this work. An example of the nanoparticles original raw spectra compared with the treated spectra and the spectra of the free porphyrin are presented as Supplementary Figure S1.

In all cases, the absorbance profile of the porphyrin remained distinctive, with a Soret band between 400 and 450 nm and Q bands observable in the 500–700 nm range. The absorbance of their Soret bands was analyzed through their intensity and their wavelength of maximum absorbance (Table 2). As previously described, except for T(PrOH)PyP, the Soret band of the porphyrins in PB pH 7 showed a red shift, between 3 and 16 nm, when compared with the free porphyrins in DMF. Additionally, a decrease in the absorption coefficient was observed, showing between 12 and 78% of their original absorbance, with all the neutral porphyrins, namely THPP, T(OAc)PP and ZnTHPP, being below 20%. Meanwhile, T(MAP)PP, a cationic porphyrin, conserved the intensity of its Soret band, with 78% of the original value. The decrease in absorbance and the red-shift observed could be due to the difference of solvent, as well as the consequence of the formation of J-aggregates because of π–π stacking between porphyrins [29,30]. As the case of the cationic porphyrins, especially for T(PrOH)PyP, their solubility in aqueous media is superior than for the other porphyrins, supporting the hypothesis of aggregates formation for the neutral compounds.

When the absorption coefficient of the Soret band of the free porphyrins in aqueous media was compared with their respective value for encapsulated porphyrins, it was generally observed that the encapsulation presents an advantage over the delivery of the free porphyrin, as encapsulated porphyrins have higher absorption coefficients than the free porphyrins in aqueous media. The exception for this rule is, once again, T(PrOH)PyP, as this cationic porphyrin has a better absorption coefficient in aqueous media, possibly due to the presence of the four hydroxyl moieties that enable the formation of hydrogen bonds with water. The Soret band wavelength of encapsulated porphyrins, suspended in PB pH 7, were neither the same than the free porphyrin in DMF nor in PB pH 7 with 5% of DMF. This suggests that aggregation of porphyrins is not the sole factor that affects the absorption of porphyrins, but also the encapsulation process modifies the absorption of the porphyrins.

**Figure 2:**
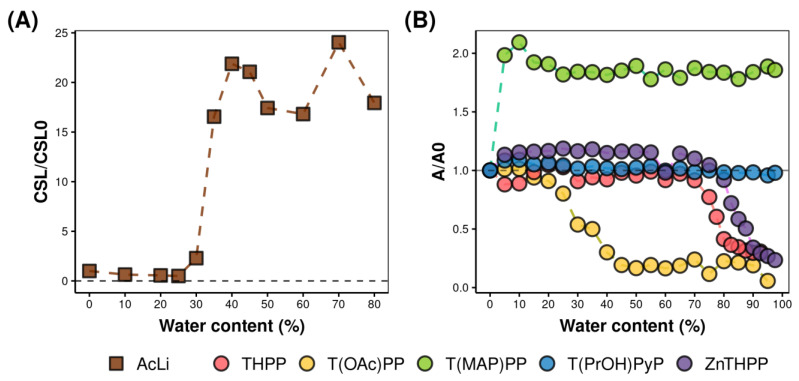
Simulation of nanoparticles formation. (**A**) Corrected scattered light (CSL) of an acetylated lignin solution (acetone, 2 mg/mL) as a function of water addition. (**B**) Porphyrin aggregation as a function of water addition. Porphyrins were dissolved in the organic solvent or solvents mixture used as the starting solution for nanoparticles formation, at a final concentration of 5 µM. Porphyrin aggregation was monitored through the maximum absorbance of the Soret band in their respective solvent: THPP, acetone; T(OAc)PP, acetone:DMF 9:1; T(MAP)PP, acetone:DMSO; T(PrOH)PyP, acetone:DMSO 9:1; ZnTHPP, THF.

In silico simulations of nanoparticles formation suggest that, when water content reaches a threshold concentration, it triggers the spontaneous formation of acetylated lignin nanoparticles [31]. However, the effect that water introduction has over the porphyrin aggregation and how it correlates with nanoparticles formation has been unexplored. In order to gain further insight into this process, acetylated lignin and porphyrins were dissolved in the solvent or solvents mixtures routinely used for the formation of loaded-porphyrin acetylated lignin nanoparticles (Table 7). Acetylated lignin aggregation, as increase in corrected scattered light, and porphyrin aggregation, as diminishment of Soret band’s absorption, were followed up as a function of water addition into the solvent mixture (Figure 2).

These observations showed that the acetylated lignin nanoparticles formation starts at a critical water concentration (CWC) of around 30%, an inflexion point where corrected scattered light increases, which correlates with previous reports [31]. On the other hand, porphyrin aggregation was assumed to be indicated by a decrease in absorbance in the Soret band upon water addition (Supplementary Figure S2). In the case of non-cationic porphyrins THPP, T(OAc)PP and ZnTHPP, water addition into the starting solution led to a widening of the Soret band, in addition to a decrease in absorbance. Meanwhile cationic porphyrins solutions showed an unaltered profile. Similarly to AcLi collapsing, non-cationic porphyrins reached a critical water concentration, as the inflexion point where absorbance suddenly decreases, indicating the aggregation point of porphyrins. The CWC for each porphyrin was different and structure-dependent. As an example, for T(OAc)PP, a porphyrin without hydroxyl moieties, its CWC was found at around 10%, while THPP and ZnTHPP, both with four free hydroxyl moieties, CWC is found at 70 and 75%, respectively. The increase in the aggregation tolerance is likely due to the presence of the hydroxyl moieties, which favors their partial solubility in aqueous media. The blockage of their hydroxyl moieties seems to provoke the rapid aggregation of T(OAc)PP in the presence of water. Meanwhile, cationic porphyrins do not aggregate upon water addition. This was expected, as both porphyrins are water-soluble, but, while absorbance of T(PrOH)PyP is not altered by water addition, T(MA)PP doubles its absorbance when water is added, suggesting that the mixture of acetone and DMSO does not favor its proper solubility. These observations suggest that encapsulation of porphyrins can happen either with the molecules as monomers (cationic porphyrins) or when porphyrins are aggregated polymers (T(OAc)PP). As result, porphyrins with a higher tolerance for water addition (T(MAP)PP > T(PrOH)PyP > ZnTHPP > THPP > T(OAc)PP) also have a better absorbance ratio, when compared to the encapsulated and free porphyrins (T(MAP)PP > ZnTHPP > THPP > T(OAc)PP) > T(PrOH)PyP). The exception of this observation is T(PrOH)PyP, as their absorbance diminishes to only 15% of its original absorbance (Table 2).

Although aggregation of porphyrins has an important role in the diminishing of light absorption, it appears that the encapsulation process per se affects the porphyrins’ ability to absorb light, with the most evident effect being light dispersion of the nanoparticles suspension. In the literature, there are several reports where formulations of photosensitizers present affected photophysical properties [32], representing a challenge for the formulation of photosensitizers.

#### 2.4.2. Fluorescence Quantum Yield

Fluorescence of porphyrins was analyzed as a function of their encapsulation. Emission spectra were obtained upon excitement at 425 nm (Supplementary Figure S3), using tetraphenyl porphyrin (TPP) in toluene as a reference [33] to calculate the fluorescent quantum yield Φ_F_ (Table 3).

In all cases, once porphyrins were encapsulated, they kept their distinctive emission spectra, with two main emission bands between 600 and 800 nm (Supplementary Figure S3). As previously explored, porphyrins in aqueous media greatly aggregated, leading to fluorescence quenching. Aggregation of porphyrins, due to high concentrations or due to water presence, is a common issue that leads to quenching of fluorescence emission and, most importantly, singlet oxygen production. The encapsulated porphyrins succeeded at delivering the porphyrins in an aqueous media, while improving their fluorescence, when compared with obtained values of the free porphyrins in PB pH 7. Thus, the encapsulation inside acetylated lignin nanoparticles aids in delivering non-cationic porphyrins into aqueous media, while preserving their fluorescence.

Although all the porphyrins kept their emission spectra bands, in some cases, there were changes in their profiles. The most relevant changes were monitored for THPP emission, which in DMF presents a major band at 663 nm, and a secondary band at 733 nm. Indeed, when THPP is encapsulated, its relative intensity of the two emission bands inverted, with a higher emission band at 733 nm, instead of 663 nm, for the encapsulated porphyrin. This has been explained by the formation of the protonated species THPPH_2_^2+^ [29], which suggests that the microenvironment inside acetylated lignin nanoparticles is acidic [20]. Further analysis (Supplementary Figure S4) revealed that encapsulated ZnTHPP and T(PrOH)PyP have similar emission profiles, with the principal emission band was found after 700 nm; this profile does not fluctuate even upon pH change in the media. Furthermore, a red-shift was observed for the excitation spectra of the “acidic” bands, when compared with the excitation spectra of the free porphyrins in DMF, and corresponding to the excitation spectra of the free porphyrins in acidic pH. Interestingly, this behavior was not found for T(OAc)PP and T(MAP)PP, as their principal emission bands were found at around 650 nm, as found in the emission spectra for the free porphyrins in DMF. These observations are structure-dependent, as THPP, T(PrOH)PyP and ZnTHPP have four free hydroxyl groups, susceptible to changes upon pH change. As a contrast, both T(OAc)PP and T(MAP)PP have blocked their hydroxyl moieties and seem to be less prone to structural changes upon pH alteration. Consequently, the structural differences between the porphyrins used in this study has permitted the elucidation of the internal environment of acetylated lignin nanoparticles, an environment that seems slightly acidic, confirming our previous observations [20].

#### 2.4.3. Singlet Oxygen Production

**Figure 3:**
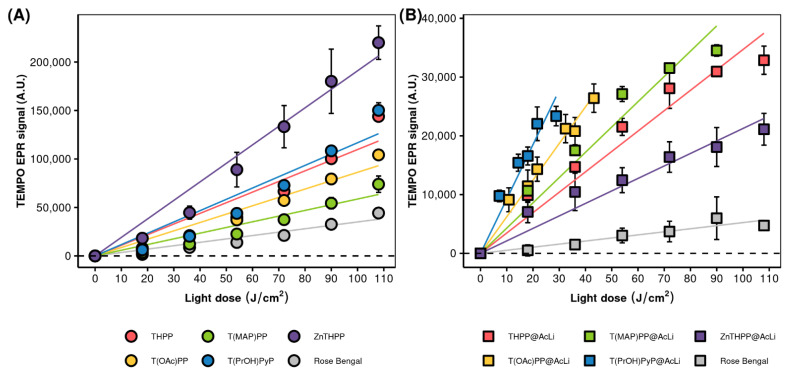
Singlet oxygen production, detected as TEMPO formation through EPR for (**A**) free porphyrins and (**B**) encapsulated porphyrins, using 12.5 mM of TEMP, either in DMF or in PB pH 7. Free porphyrins were dissolved in DMF, for a final concentration of 1.5 µM, while encapsulated porphyrins were suspended in PB pH 7, at 25 µM of their corresponding porphyrin. Rose Bengal was used as a standard, at (**A**) 1.5 µM and (**B**) 5 µM. The samples were irradiated with blue LED-light (450–460 nm, 60 mW/cm^2^), at different light doses. The results shown are the average of three independent experiments.

Fluorescence emission is not the only photophysical feature of importance for those porphyrins, as these molecules work as excellent photosensitizers, producing singlet oxygen upon light irradiation [34]. Singlet oxygen production of porphyrin-loaded nanoparticles was monitored through electron paramagnetic spectroscopy (EPR), in combination with the use of a singlet oxygen trap, 2,2,6,6-pyperidine (TEMP), as already reported in the literature [20,35]. TEMP easily reacts with singlet oxygen to form 2,2,6,6-tetramethyl-1-piperidinyloxy (TEMPO), a stable radical that can be detected through EPR. Singlet oxygen production was driven through irradiation of the samples with blue LED-light (450–460 nm, 60 mW/cm^2^) through increasing light doses (Figure 3). The tendencies of the singlet oxygen production were compared with the singlet oxygen quantum yield of the free porphyrins (Table 4), obtained through measuring the singlet oxygen phosphorescence decay with near-infrared spectroscopy (NIRS), using phenalenone as standard (Φ_Δ_ = 1.0 [36]) and excitation at 355 nm (Supplementary Figure S5).

Singlet oxygen quantum yields for the free porphyrins in DMF showed that our most efficient photosensitizer is ZnTHPP (Φ_Δ_ = 0.7320), followed by T(OAc)PP and T(MAP)PP and lastly by THPP and T(PrOH)PyP. The results obtained with EPR for the free porphyrins dissolved in DMF contrast with the results obtained through NIRS, as the singlet oxygen production efficiency is shown as ZnTHPP > T(PrOH)PyP > THPP > T(OAc)PP > T(MAP)PP. These discrepancies between EPR and NIRS obtained values have been previously explored [39], and it has been demonstrated that TEMP can act as a quencher of the excited triplet state of certain photosensitizers, leading to an overestimation of the production of singlet oxygen. Nevertheless, NIRS could not be used for the detection of singlet oxygen quantum production for the encapsulated porphyrins, not even in deuterated water. Thus, EPR was used for the detection of singlet oxygen production of encapsulated porphyrins, and the results show that singlet oxygen production tendency goes T(PrOH)PyP@AcLi > T(OAc)PP@AcLi > T(MAP)PP@AcLi > THPP@AcLi > ZnTHPP@AcLi. Interestingly, these results coincide with the fluorescent quantum yield of the encapsulated porphyrins, with T(PrOH)PyP@AcLi and ZnTHPP@AcLi having the highest and lowest fluorescent quantum yield, respectively (Table 3). This suggests that encapsulation affects both singlet oxygen and fluorescence in the same magnitude, as could be expected.

Something remarkable is that we were able to find singlet oxygen production at all our nanoparticles, similarly to our previous report [20]. Lignin has been rarely used as a material on PDT and PACT applications, due to its well-known antioxidant activity, but a report indicates that esterified lignin is able to produce singlet oxygen upon light irradiation [40]. However, other reports also specify that, even if lignin is able to produce singlet oxygen, the amount produced by the non-loaded nanoparticles @AcLi is insignificant when compared with the singlet oxygen produced by the porphyrins [20]. Here, we provide evidence that several types of porphyrins, once encapsulated inside acetylated lignin nanoparticles, are still able to generate singlet oxygen. Additionally, it has been hypothesized that porphyrins remain inside the nanoparticles and that only the singlet oxygen is diffusing through and towards the outside of the nanoparticle

#### 2.4.4. Photobleaching

**Figure 4:**
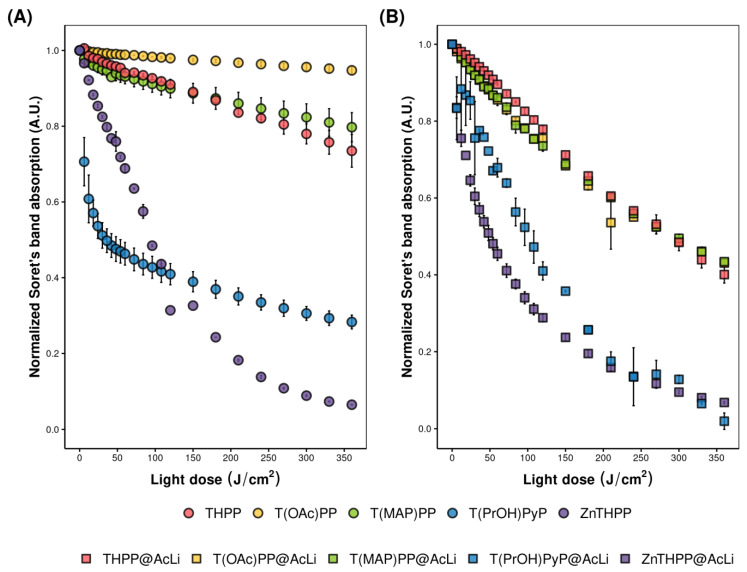
Photobleaching of (**A**) free porphyrins and (**B**) encapsulated porphyrins. Free porphyrins were dissolved in DMF at 5 µM, while nanoparticles were suspended in PB pH 7, at 5 µM of their respective porphyrin. Three milliliters of both solutions were deposited in an open quartz square cell of 1 cm width, with constant stirring, under blue-LED light irradiation (100 mW/cm^2^), under several lapses. The normalized absorbance is presented and the results presented are the average of two independent experiments.

The production of reactive oxygen species can lead to the self-destruction of the photosensitizer. Previous reports indicate that THPP encapsulated inside acetylated lignin nanoparticles does not photobleach over white-LED light irradiation [20]. In order to deepen insight into the behavior of porphyrins encapsulated into acetylated lignin nanoparticles, the destruction of the porphyrins, as a loss of absorbance at the Soret band, was followed up for both free porphyrins (Supplementary Figure S6) and their encapsulated formulations (Supplementary Figure S7), under blue-LED light irradiation, at several light dosages (Figure 4).

**Scheme 2:**
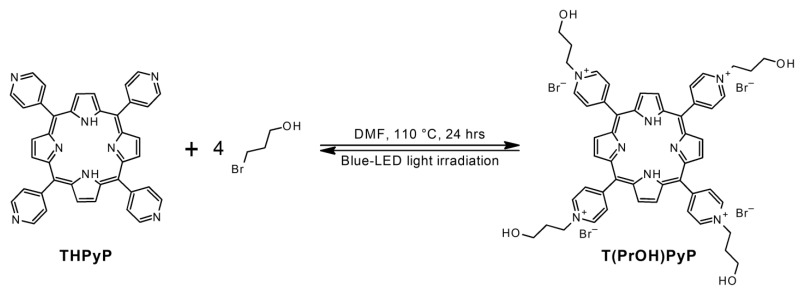
Synthesis of T(PrOH)PyP, from THPyP. When T(PrOH)PyP is irradiated with blue light, it seems to be destroyed back to THPyP.

In most cases, porphyrin degradation was easily followed up at the Soret band, with porphyrins profile remaining similar through the whole irradiation process. However, in the case of T(PrOH)PyP and T(PrOH)PyP@AcLi, since the beginning of irradiation, there was a blue shift of the Soret and Q bands, leading to a fast disruption, followed up by a slower degradation. This could be indicative of the destruction of aggregates of porphyrins, as it has been previously observed that T(PrOH)PyP solubility is not favored in DMF (Table 2). Nevertheless, when the spectra of T(PrOH)PyP is compared with the spectra of THPyP, the starting porphyrin for T(PrOH)PyP, a match was found between the photobleached T(PrOH)PyP and THPyP (Supplementary Figure S8). Then, blue light irradiation could be led to the fast degradation of T(PrOH)PyP to THPyP, which has a slower rate of photodegradation and worst solubility in DMF (Scheme 2). This is similarly observed in T(PrOH)PyP nanoparticles.

Previous reports indicated that THPP@AcLi, when exposed to white light in static conditions, withstood photodegradation [20]. In this report, we present data with a different experimental approach, as light irradiation is five times higher, using a narrow-band light source and with constant stirring that ensures both aeration and prevents nanoparticles precipitation. Under these conditions, it was found that encapsulated porphyrins degrade at a similar or higher speed than the free porphyrins. This could be due to the high concentration of porphyrins in the nanoparticles volume, leading to an easier self-destruction. Interestingly, THPP@AcLi, T(OAc)PP@AcLi and T(MAP)PP@AcLi have similar photobleaching rates when encapsulated, unlike when they are dissolved in organic solvent. This is interesting, as the singlet oxygen production is greatly reduced for the encapsulated porphyrins, and then a lower photobleaching would be expected. This finding corroborates our hypothesis of a singlet oxygen diffusion through the nanoparticle, creating a high concentration of singlet oxygen inside the nanoparticles and leading to an accelerated rate of photobleaching for the encapsulated porphyrins.

**Figure 5:**
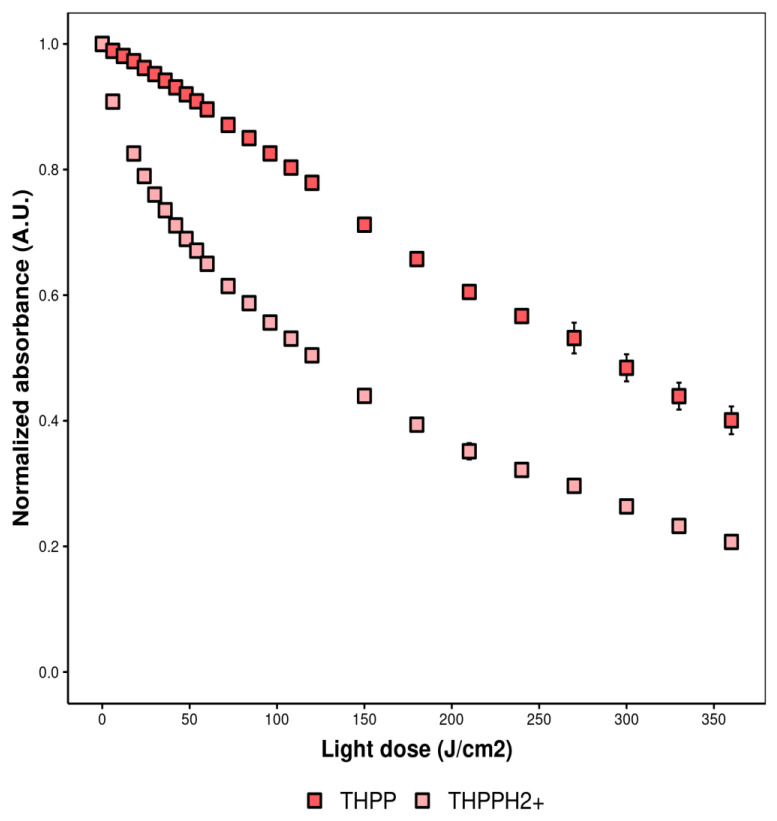
Compared photodegradation of THPP and THPPH_2_^2+^, as both co-exist inside acetylated lignin nanoparticles, with the cationic species degrading faster upon blue-LED light irradiation.

On the other hand, it was previously described that THPP@AcLi presents a Soret band and a B-band, which corresponds to the protonated form of THPP, THPPH_2_^2+^. This B-band was described to degrade at a faster pace than the main Soret band and to have a great contribution on the fluorescence of the nanoparticles [20]. Our results so far indicate a similar trend, with the B-band degrading at a faster pace than the Soret band, until reaching the base of the peak of THPP (Figure 5). This suggest that THPPH_2_^2+^ could have an important role on ROS production, as it does in fluorescence, even though it has a short half-life.

### 2.5. Effect of the Aqueous Media on Porphyrins and Porphyrin Loaded Nanoparticles

Through all the present work, the pH of the medium has played an important role at the different photophysical parameters of our nanoparticles. To corroborate and elaborate this aspect, the influence of the pH on the medium for the free porphyrins and the encapsulated porphyrins was analyzed. In the literature, there are some examples where drug-loaded lignin nanoparticles are able to release their cargo into the media, in acidic pH [41,42,43,44]. However, porphyrins loaded into acetylated lignin nanoparticles seem to be unaltered when the pH of the media fluctuates [20], and this could be due to a diminishing of the accessibility of the solvent during acetylated lignin nanoparticles formation [40], which could eventually lead to the permanent entrapment of porphyrins inside nanoparticles.

#### 2.5.1. Effect of Fluctuations of pH into the Medium

**Figure 6:**
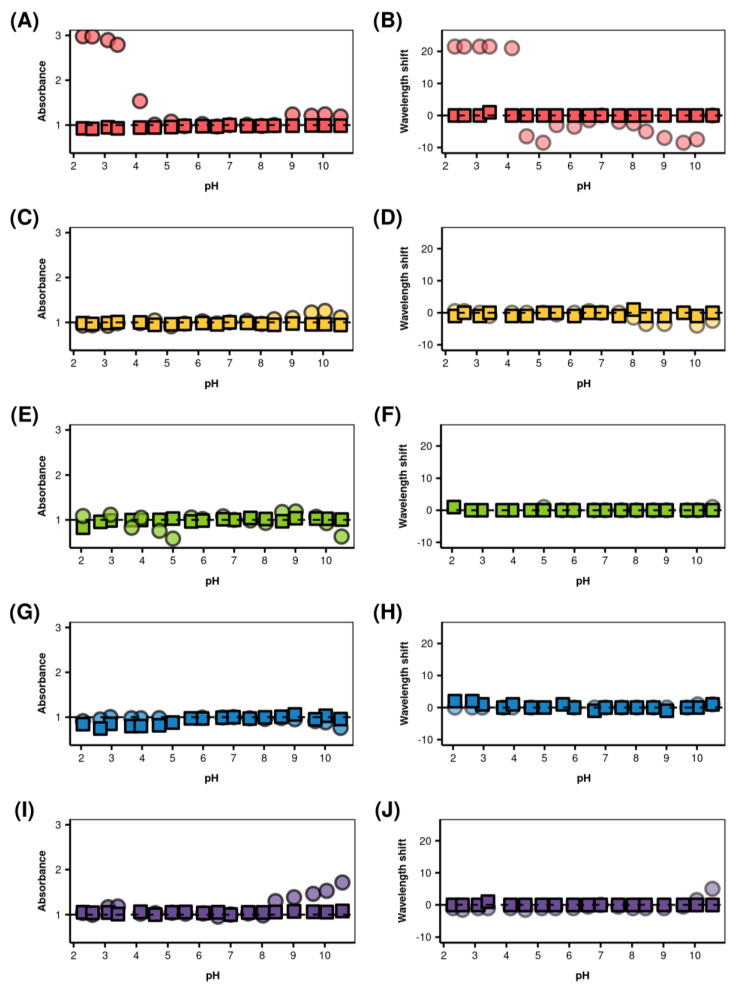
Effect of pH on the UV-vis absorption of (**A**) THPP, (**C**) T(OAc)PP, (**E**) T(MAP)PP, (**G**) T(PrOH)PyP and (**I**) ZnTHPP, as free porphyrins (circles) or as encapsulated poprhyrins (squares). The effect on the Soret band maximum wavelength was also analyzed, for (**B**) THPP, (**D**) T(OAc)PP, (**F**) T(MAP)PP, (**H**) T(PrOH)PyP and (**J**) ZnTHPP, as free porphyrins (circles) or as encapsulated porphyrins (squares). Free porphyrins were dissolved in DMF and diluted in aqueous media to a final concentration of 5 µM and 5% DMF; encapsulated porphyrins were suspended in aqueous media, for a final concentration of 5 µM, of their corresponding porphyrin. Data were normalized, as based on the obtained absorbance and Soret’s band wavelength at pH 7.

It has been hypothesized that, inside the nanoparticles, an acidic media permits the formation of THPPH_2_^2+^, the protonated derivative of THPP, which appears to have an important role on the fluorescence, singlet oxygen formation and photobleaching. To demonstrate if these observations are extensive to other porphyrins encapsulated inside nanoparticles, the influence of pH in the media for both free and encapsulated porphyrins was analyzed (Figure 6).

As previously reported, encapsulation of porphyrins inside acetylated lignin nanoparticles prevents changes of the porphyrins, as a result of pH changes. This is particularly evident when comparing the spectra of the free and encapsulated porphyrins, with the latest remaining unaltered through the pH changes (Supplementary Figure S9). This corroborates our previous reports, supporting our hypothesis of diminished solvent accessibility for the porphyrins, once inside the nanoparticles.

#### 2.5.2. pH Driven Release

**Figure 7:**
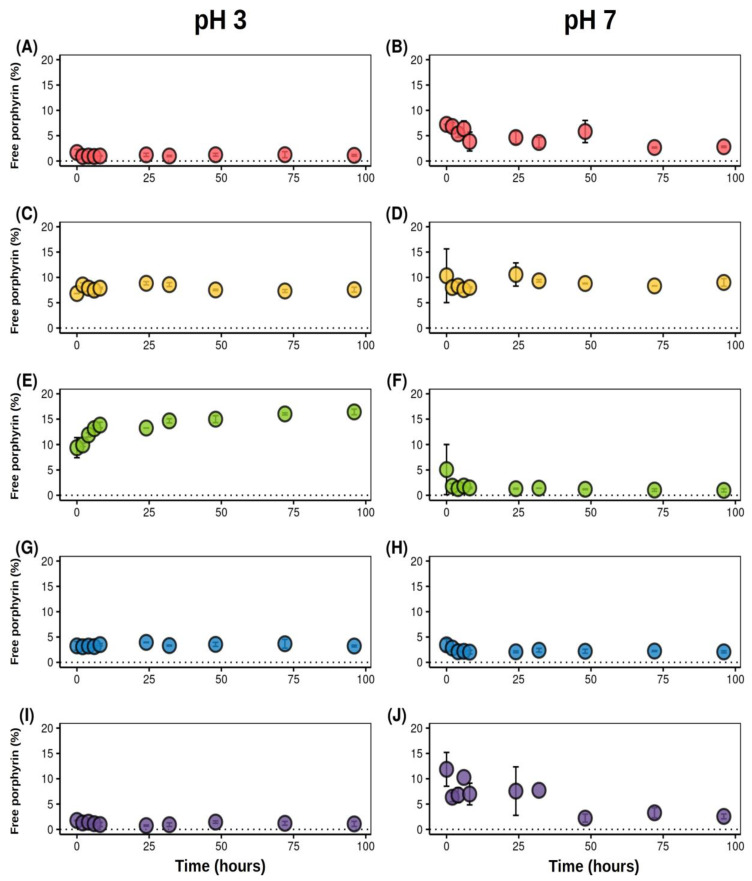
Leaking of porphyrins over time, as a function of pH. Porphyrin leakage was evaluated in pH 3 and pH 7 for THPP (**A,B**), T(OAc)PP (**C**,**D**), T(MAP)PP (**E**,**F**), T(PrOH)PyP (**G**,**H**) and ZnTHPP (**I**,**J**), respectively. The leakage is presented as free porphyrin, with respect to the initial amount of porphyrin, 20 µM.

The lack of interaction between the medium and the encapsulated porphyrins could mean that the porphyrins are not released into the media, even when a pH change occurs. In the literature, there are reports that indicate that lignin nanoparticles loaded with benzazulene [41,42], sorafenib [42], 10-hydroxycamptothecin [43] and emamectin benzoate [44] are able to release their cargo in acidic pH. Furthermore, some reports indicate that release happens even at neutral pH, but is enhanced in acidic pH [41]. However, none of these reports were done in acetylated lignin, but in lignin chemically modified with functional groups that are susceptible to pH changes, as carboxyl and histidine moieties. Nevertheless, the capacity of our nanoparticles to leak the compound in response to an acidic pH was analyzed (Figure 7).

Nanoparticles were suspended either at pH 3 or pH 7 buffer and at each time a sample was taken and centrifuged, sampling over the supernatant obtained. The supernatants were analyzed through fluorescence and reported as free porphyrin, with respect to the initial amount of porphyrins (20 µM). Interestingly, we could observe differences between the amount of free porphyrin at time zero as a function of pH, for THPP and ZnTHPP. Both samples showed a higher amount of free porphyrin when suspended in pH 7. Nevertheless, this namely free porphyrin quickly degrades and at the final time of observation there could be no distinguishable differences as a function of pH.

For most of the cases, the amount of free porphyrin remained stable from the beginning to the end of the experiment (Supplementary Table S1). Remarkably, besides the decrease in free porphyrin found for THPP and ZnTHPP, it was found that the amount of free T(MAP)PP increased significantly from 9.38 to 16.4% when the nanoparticles were suspended in pH 3 buffer. Furthermore, this effect was not found for the same nanoparticles when suspended in pH 7. Similar behavior could not be found in the other porphyrins, not even in T(PrOH)PyP, which shares T(MAP)PP’s cationic nature. Our previous report has indicated that THPP@AcLi would keep its porphyrin for up to 60 days, with minimal leaking (9%). Although the present approach lasted a shorter time, this was a more dynamic experiment, as nanoparticles suspensions were under constant stirring, ensuring a better interaction with the media. Nevertheless, we were not able to observe leaking in any other than for T(MAP)PP in acidic pH.

The tight entrapment of porphyrins inside nanoparticles is interesting as an immobilization vehicle for porphyrins. Normally, to ensure such a tight interaction with a material, covalent bonds are used, increasing the cost of the developed technology, but ensuring their lasting, and even providing molecular targeting [45]. Our nanoparticles then provide an effective and endurable strategy, without the nuisance of chemical modifications to prevent leakage from the material.

### 2.6. Photodynamic Antimicrobial Chemotherapy Effect

Our latest report indicated that THPP@AcLi was able to eradicate three strains of Gram-positive bacteria, while remaining ineffective against Gram-negative bacteria, using white-LED light irradiation as a light source. This was a breakthrough, as it was demonstrated that nanoparticles with low apparent singlet oxygen production were able to kill bacteria using concentrations below 1 µM. The challenge was set then to eradicate Gram-negative bacteria as well, bacteria which are normally impermeable to porphyrins, but which are sensitive to cationic photosensitizers.

#### 2.6.1. Bacteriostatic Effect

The bacteriostatic effect of both free and encapsulated porphyrins was tested against *Staphylococcus aureus* and *Escherichia coli*, as Gram-positive and Gram-negative models, respectively. The minimal inhibitory concentrations (MIC) for the light and dark conditions are shown in Table 5, while the obtained graphs of growth as a function of concentration can be found in Supplementary Figures S10 and S11.

In the conditions tested, only the free porphyrin T(OAc)PP was unable to arrest the growth of *S. aureus,* while only the free cationic porphyrins were able to arrest the growth of *E. coli*. However, all the encapsulated porphyrins seemed to be unable to inhibit the growth of both *S. aureus* and *E. coli*. Nevertheless, these results, which contrast with our previous report, could be due to a methodological difference. In our last report, bacteria were grown for 6 h at 37 °C after the light treatment, before measuring the resultant growth. However, experimental limitations due to COVID-19 restrictions prevented from observing bacterial growth in a narrower time window, monitoring bacteria growth at 16 h after light irradiation. Then, any lack of growth can be due a total annihilation of bacteria due to a photodynamic effect, or due to a slow, dark chemotoxic effect. Our previous observations indicated that THPP@AcLi were unable to produce dark toxicity in Gram-positive bacteria strains [20], and thus the high MIC values for the nanoparticles could be camouflaged by a bacterial recovery and low dark toxicity.

#### 2.6.2. Bactericidal Effect

The obtained MIC results were complemented with the minimal bactericidal concentration (MBC), understood as the concentration with at least three logarithmic orders of magnitude reduction in the bacterial counting. When compared, MBC are expected to be several orders of magnitude lower than MIC. Normally, in chemotherapy, this is the other way around, with MBC > MIC, but this is considering that the chemotoxic effect is always present and rarely disappears over time. In the case of photodynamic compounds, normally after light irradiation, bacteria cells are cultured and able to recover after the ROS production driven by light, so it is not surprising that obtained MIC values are overestimations of the actual concentrations needed to kill bacteria. Here, we present the MBC values obtained for *S. aureus* (Supplementary Figure S12) and *E. coli* (Supplementary Figure S13), after blue-LED light irradiation (Table 6).

As expected, the MBC values found were lower than the MIC values, indicating that most of the bacteriostatic effect observed in the MIC values are due to the light-driven bactericide effect. Additionally, the MBC values for all the treatments were lower for the irradiated samples than for the non-irradiated bacteria, and then bacterial death results in a photodynamic effect.

Interestingly, while free T(OAc)PP fails to diminish the bacterial concentration of *S. aureus*, the nanoparticles T(OAc)PP@AcLi were able to diminish in ~2.5 log the bacterial concentration of *S. aureus* with 12.5 µM of the porphyrin. Surprisingly, this was the best result obtained, with an increase in concentration failing to further diminish the bacterial concentration of *S. aureus*. Similar results were observed for T(MAP)PP@AcLi and ZnTHPP@AcLi with an increase in bacterial survival on the highest concentrations tested, 50 µM. This effect is likely to be due to the high concentration of nanoparticles, which may provoke light diffraction, preventing nanoparticles and bacteria from the bottom being reached by the light. Consequently, it is likely that this problem could be overcome with stirring of the sample, which was not done due to the short irradiation times and the small volume of the samples (30 s and 100 µL). This effect is not observed for THPP@AcLi and T(PrOH)PyP@AcLi, as their MIC values are 0.7813 and 1 µM, respectively, and the presence of lignin and light dispersion is neglected. Nevertheless, it is remarkable that T(OAc)PP@AcLi was able to diminish the bacterial concentration, where the free T(OAc)PP failed to do so. As previously explored throughout this report, T(OAc)PP is the most lipophilic porphyrin, and greatly aggregates in aqueous media. Then, through our approach we were able to deliver a lipophilic porphyrin and provide it with an antibacterial activity against Gram-positive bacteria.

In general, the encapsulation of porphyrins diminished their efficiency, when compared with the free porphyrins in aqueous media, despite its aggregated character. Nevertheless, encapsulated porphyrins were unable to diminish the bacterial concentration when incubated in the dark. This was previously observed for THPP@AcLi, where null dark toxicity was found for several Gram-positive strains [20]. In this report, it was shown that THPP and ZnTHPP, are able to diminish the bacterial viability after light irradiation (MBC 0.0488 and 0.0977 µM, respectively), but they also diminish the bacterial viability in the dark, with reductions of 2.5 and 1.4, respectively, at the same concentrations. One of the most appreciated characteristics of PACT is its activation upon light irradiation, which permits modulation and control of the bactericidal effect, keeping at bay any antibacterial resistance which may arise. Thus, the presence of dark toxicity is pernicious to any research related to PACT. Our formulation presents an advantage over the use of the free porphyrins, as our formulation requires a precise light dose to exert their antibacterial activity. As an example, in the dark against *S. aureus*, ZnTHPP@AcLi is unable to diminish the bacterial survival at 50 µM, the highest concentration tested, while ZnTHPP diminishes in 3-log the bacterial survival at around 0.3 µM. Consequently, encapsulation of porphyrins prevents dark toxicity, with the latest being a consequence of chemotoxicity and likely to be unrelated to ROS production. It is remarkable that the free cationic porphyrins did not present dark toxicity for *S. aureus* or *E. coli*.

The lack of dark toxicity from our formulation also suggests that porphyrins, once entrapped inside the acetylated lignin nanoparticles, are unable to enter the cell and provoke chemotoxic damage in the absence of light, which is supported by the stability of our formulation at pH 7. This gives an insight into their killing mechanism, and it can be hypothesized that nanoparticles interact with the bacterial wall or membrane, with the resulting death being due to the localized production of singlet oxygen from the entrapped porphyrins, without requiring the entry of the porphyrins into the cell.

While most of the free porphyrins and nanoparticles were able to diminish the bacterial count of *S. aureus*, only the free cationic porphyrins were able to kill *E. coli*, as thoroughly explored in the literature [38,46,47]. Interestingly, T(MAP)PP@AcLi was unable to diminish the bacterial counts of *E. coli*. This corresponds with the results presented for *S. aureus*, where we observed a decrease of approximately 2-log, at its best. Interestingly, T(PrOH)PyP@AcLi was able to diminish 3-log of bacterial survival at 6.25 µM, and so the lack of effect of T(MAP)PP@AcLi is not due to its cationic charges, but is a consequence of another factor.

## 3. Conclusions

In this work the encapsulation of five porphyrins was analyzed, with different chemical characteristics, inside acetylated lignin nanoparticles. It was demonstrated that acetylated lignin nanoparticles are a suitable vehicle for several kinds of porphyrins, preserving their photochemical and antibacterial properties. Here, the stability of the nanoparticles was demonstrated, which seem to be unable to leak the porphyrins once encapsulated. Furthermore, it was demonstrated that, through the increase in bioavailability, a lipophilic porphyrin as T(OAc)PP was able to diminish bacterial survival. This observation is a fundamental stone for easy, cheap and green encapsulation of lipophilic tetrapyrrolic compounds, which in aqueous media greatly aggregate, quenching the production of singlet oxygen.

Although the mechanisms of cellular death driven by porphyrin-loaded acetylated lignin nanoparticles are still unclear, these observations are likely to push forward the research on lignin and lignin nanoparticles, as suitable materials for the formulation of photosensitizers against bacterial proliferation.

## 4. Materials and Methods

### 4.1. Materials, Equipment and Microbiological Strains

Kraft lignin was kindly donated by the Université du Québec à Trois-Rivières, Canada. 5,10,15,20-tetrakis(4-hydroxyphenyl)-21H,23H-porphine (THPP), 5,10,15,20-tetrakisphenyl-21H,23H-porphine (TPP), 2,2,6,6-tetramethylpiperidine (TEMP), and Rose Bengal, were purchased at Sigma-Aldrich (Lyon, France). THPyP was purchased at Porphychem (Dijon, France). Other solvents and reagents were bought at Fluorochem (Hadfield, UK), Alfa Aesar (Haverhill, MA, USA), TCI (Paris, France), Carlo Erba (Barcelona, Spain), Fisher Chemicals (Hampton, VA, USA) and VWR Chemicals (Radnor, PA, USA). All chemicals were used as received, without further purification.

The ^1^H spectra were recorded on a 400 Bruker Advance spectrophotometer or a Bruker DPX500 NMR spectrophotometer, using tetramethylsilane as an internal standard.

*E. coli* CIP 53.126 and *S. aureus* CIP 76.25 were obtained from the Institute Pasteur Collection (Institute Pasteur, Paris, France). All bacterial strains were kept frozen as small aliquots (200 µL), at −78 °C, with glycerol 50% as cryopreservant. A whole aliquot was used for each culture, avoiding defrosting of the other samples. *E. coli* was grown in Luria Bertani (LB) broth (Biokar, Allone, France; tryptone 10 g/L, sodium chloride 10 g/L, yeast extract 5 g/L), while *S. aureus* was routinely grown in trypto-casein soy medium (Biokar, Allone, France; tryptone 17 g/L, papaic digest of soybean meal 3 g/L, glucose 2.5 g/L, dipotassium phosphate 2.5 g/L, sodium chloride 2 g/L), prepared as a broth (LBB and TSB) or as a solid media (TSA; 1.7% agar) according to standard procedures. Saline solution (0.9% NaCl) and phosphate buffer pH 7 (PB pH 7, NaH_2_PO_4_•2H_2_O 0.964 g/L, Na_2_HPO_4_•2H_2_O 3.463 g/L) were routinely prepared and sterilized.

### 4.2. Synthesis of Porphyrins

#### 4.2.1. 5,10,15,20-Tetrakis (4-acetyloxyphenyl)-21H,23H-porphine

Freshly distilled pyrrole (1.2210 g, 18.2 mmol, 1 eq.) was added dropwise to a refluxing solution of propionic acid (68.5 mL) containing 4-acetoxybenzaldehyde (2.987 g, 18.2 mmol, 1 eq). The mixture was stirred under reflux for 30 min and cooled to room temperature. The reaction mixture was filtered and washed with MeOH. The crude product was purified by silica gel column chromatography (CHCl_3_/MeOH 9/1, *v*/*v*) to afford T(OAc)PP as a purple solid (yield 12.3%). ^1^H NMR (500 MHz, CDCl_3_): δ (ppm) = 8.88 (s, 8H, Hpyrr), 8.21 (d, *J* = 11.0 Hz, 8H, H-Ar), 7.52 (d, *J* = 11.0 Hz, 8H, H-Ar), 2.5 (s, 12H, CH_3_), −2.81 (s, 2H, NH).

#### 4.2.2. 5,10,15,20-Tetrakis(4-(3-*N*,*N*,*N*-trimethylammoniumpropoxy)-phenyl)-21H,23H-porphine bromine

Previously dried commercial THPP (150 mg, 0.2652 mmol, 1 eq.) were dissolved in dry DMF (5 mL), with further addition of cesium carbonate (753.1 mg, 2.3114 mmol, 8.7 eq.), under constant stirring and nitrogen atmosphere. Then, (3-bromopropyl)trimethylammonium bromide (583.1 mg, 2.2337 mmol, 8.4 eq.) was added and the reaction was heated at 100 °C for 24 h. The reaction was cooled to room temperature and precipitated with CH_2_Cl_2_ (100 mL). The obtained solid was filtrated and evaporated. The solid was dissolved in water (50 mL) and extracted with 50 mL of CH_2_Cl_2_ three times. The aqueous phase was recovered and evaporated, to afford T(PrOH)PyP as a crystalline violet solid (yield 72%). ^1^H NMR (400 MHz, DMSO-d_6_): δ (ppm) = 9.59 (d, *J* = 6.68 Hz, 8H, H-Ar), 9.23 (s, 8H, Hpyrr), 9.02 (d, *J* = 6.68 Hz, 8H, Har), 5.07 (m, 8H, CH_2_-N), 3.80 (m, 8H, CH_2_-O), 2.46 (m, 8H, CH_3_), −3.08 (s, 2H, NH).

#### 4.2.3. 5,10,15,20-Tetrakis (4-(3-hydroxy)propyloxypyridyl)-21H,23H-porphine bromine

Previously dried commercial THPyP (101.2 mg, 0.1636 mmol, 1 eq.) were dissolved in dry DMF (8 mL), under constant stirring and nitrogen atmosphere. Then, 3-bromopropanol (922 mg, 6.6350 mmol, 40 eq.) were added dropwise and the reaction was heated at 100 °C over 24 h. The reaction was cooled to room temperature and precipitated with CH_2_Cl_2_ (50 mL). The obtained solid was filtrated and evaporated. The solid was dissolved in MeOH and purified by silica gel flash column chromatography (Acetonitrile/MeOH/Water 6/2/2), to afford a T(MAP)PP as a violet powder (yield 62%). ^1^H NMR (400 MHz, DMSO-d_6_): δ (ppm) = 8.92 (s, 8H, Hpyrr), 8.21 (d, *J* = 8 Hz, 8H, H-Ar), 7.42 (d, *J* = 8 Hz, 8H, H-Ar), 4.45 (m, 8H, CH_2_-O), 3.76 (m, 8H, CH_2_-N), 3.29 (s, 36H, CH_3_), 2.36 (m, 8H, CH_2_), −2.83 (s, 2H, NH).

#### 4.2.4. Zinc (II) 5,10,15,20-Tetrakis (4-hydroxyphenyl)-21H,23H-porphine

Commercial THPP (60 mg, 0.09 mmol, 1 eq.) was dissolved in DMF anhydre (10 mL), with further addition of zinc acetate (0.1 g, 0.44 mmol, 5 eq.). The mixture was stirred under reflux under a nitrogen atmosphere, over 1.5 h and cooled to room temperature. The reaction mixture was then precipitated in distilled water over 12 h, at 4 °C. The solid was filtrated, obtaining ZnTHPP as a violet powder solid (yield 84%). ^1^H NMR (500 MHz, DMSO-d_6_): δ (ppm) = 9.81 (s, 4H, Ar-OH), 8.88 (s, 8H, Hpyrr), 7.95 (d, *J* = 8.28 Hz, 8H, H-Ar), 7.17 (d, *J* = 8.36, 8H, H-Ar).

### 4.3. Preparation and Quantification of Porphyrin-Loaded Acetylated Lignin Nanoparticles

Nanoparticles were prepared using acetylated lignin [30]. Acetylated lignin (2 mg/mL) and porphyrins (0.2 mg/mL) were dissolved in a solvent or mixture solvent, accordingly to the porphyrins’ solubility (Table 7). The starting solutions were dialyzed on a regenerated cellulose membrane rod (Fisherbrand, Ottawa, Canada; 12–14 KDa), against distilled water, at room temperature and without stirring, for 24 h. After dialysis, nanoparticles were centrifuged at 10,000× *g* for 30 min and washed twice with distilled water. Nanoparticles were suspended in distilled water and kept in the dark at room temperature until further use. For quantification, nanoparticles were diluted and quantified, using their absorption coefficients, in acetone (THPP (ε_419 nm_ = 388,500 M^−1^cm^−1^) and ZnTHPP (ε_425 nm_ = 485,914 M^−1^cm^−1^)), DMSO (T(MAP)PP (ε_423 nm_ = 242,207 M^−1^cm^−1^)) or DMF (T(OAc)PP (ε_418 nm_ = 388,725 M^−1^cm^−1^) and T(PrOH)PyP (ε_426 nm_ = 132,376 M^−1^cm^−1^)).

### 4.4. Physical Characterization

Nanoparticles size was analyzed through DLS on a Zetasizer Nano-ZS (Malvern Instrument, Malvern, UK). For each sample, three measurements were performed on each sample, using a disposable plastic cuvette, at 20 °C and using a light scattering angle of 173° and a refractive index of 1.61, for lignin materials [48]. The obtained DLS raw data were fitted to a Gaussian distribution model, excluding the values with less than 1% of frequency. The normality of the obtained model was evaluated through its R square coefficient and through the analysis of the residuals, with a D’Agostino-Pearson Omnibus K2 test, affording the mean size (geometrical mean) and the standard deviation (σ), which allowed us to approximate the range where 95% of the nanoparticles were found (2σ).

Zeta potential was measured using a DTS1070 cuvette (Malvern Instrument, Malvern, UK), in a Zetasizer Nano-ZS (Malvern Instrument, Malvern, UK), obtaining five measurements for each sample.

### 4.5. Photophysical Characterization

UV-vis spectra were recorded on a Hitachi U-2001 (Tokyo, Japan) or Analytic Jena Specord 210 (Jena, Germany). Spectra were collected from 350 up to 800 nm, using standard quartz cuvettes of 1 cm of optical path.

The fluorescence quantum yield was calculated as described elsewhere [11,20]. Fluorescence spectra were recorded in a Horiba Scientific Spectrofluorometer Fluoromax-4 (Potsdam, Germany) or in an Edinburgh Instruments FLS980 (Edinburgh, UK). Spectra were collected from 550 up to 800 nm, using standard quartz cuvettes of 1 cm of optical path, at room temperature. Fluorescence quantum yields (Φ_F_) were calculated using TPP dissolved toluene in a reference (Φ_F_ = 0.11), by comparing the area of the integrated fluorescence of the samples (F_s_) with the fluorescence of the reference (F_ref_) and corrected by the absorption of the sample (A_s_) and the reference (A_ref_) at the excitation wavelength and by the refractive index of the solvents used for the sample (η_s_) and reference (η_ref_) solutions (Equation (1)).

(1) $$\Phi_{F}=\Phi_{F}^{\mathrm{ref}}\frac{F_{s}A_{\mathrm{ref}}\eta_{s}^{2}}{F_{\mathrm{ref}}A_{s}\eta_{\mathrm{ref}}^{2}}$$

Singlet oxygen phosphorescence decay was monitored at room temperature, according to previously described procedures [11]. Porphyrins were dissolved in DMF, until they reached an optical density of 0.18. The porphyrins solutions were excited at 355 nm, using an Nd-YAG laser Spectra-Physics Quanta-Ray GRC-130 (Stahnsdorf, Germany). The singlet oxygen phosphorescence was collected at 1270 nm, in a Hamamatsu R5509-42 (Hamamatsu, Japan) photomultiplier, cooled to 193 K in a liquid nitrogen chamber, after selection of the wavelength with a monochromator with 600 lines grading. A Newport filter model 10LWF-1000-B was used in the emission to prevent light scattering and fluorescence. As a reference for the singlet oxygen production, phenalenone was used (Φ_δ_^ref^ = 1.0) [35]. The decays obtained were extrapolated to time-zero, obtaining the initial emission intensities as a function of laser intensity. The singlet oxygen quantum yields were obtained by comparing the linear dependence of the singlet oxygen emission and the energy of the laser pulse for the sample (S_s_) and the reference (S_ref_), corrected by the absorption of the sample (A_s_) and reference (A_ref_) at the excitation wavelength, considering the singlet oxygen quantum yield of the reference (Equation (2)).

(2) $$\Phi_{\Delta }=\Phi_{\Delta }^{\mathrm{ref}}\frac{S_{s}1-10^{-A_{\mathrm{ref}}}}{S_{\mathrm{ref}}1-10^{-A_{s}}}$$

Singlet oxygen production detected through EPR were recorded as described elsewhere [20,35]. The samples were exposed to a blue-LED light (LED-Illuminator, Luzchem, Gluocester, Canada); light irradiation was measured with a handheld powermeter (Lasercheck, Coherent, Santa Clara, CA, USA), centered at 455 nm. EPR spectra were recorded with a Bruker Model ESP300E spectrometer (Berlin, Germany) operating at room temperature. Routinely, a fresh solution of 25 mM 2,2,6,6- tetramethylpiperidine (TEMP) was prepared in DMF or PB pH 7. Free porphyrins were dissolved in DMF and diluted in DMF until they reached 3 µM. Nanoparticles suspensions were diluted at 50 µM in PB pH 7. For singlet oxygen detection, 50 µL of the fresh TEMP solution were mixed with 50 µL sample to analyze. The solution obtained was immediately transferred into quartz capillaries (100 µL) and placed at 14.5 cm from the source of illumination with a light intensity irradiation of 60 mW/cm^2^, for appropriate periods of time. A dark control was prepared, and Rose Bengal in DMF or PB pH 7 was used as a standard. EPR spectra were performed under the following conditions: modulation frequency, 100 kHz; microwave frequency, 9.78 GHz; microwave power, 4 mW; modulation amplitude, 0.987 G; time constant, 10.24 ms; scans number, 2.

Photobleaching experiments were done as stated elsewhere [11]. Free porphyrins were dissolved in DMF, while the encapsulated porphyrins were suspended in PB pH 7, both at a final concentration of 5 µM. A volume of 3 mL was deposited in a standard quartz cuvette of 1 cm of optical path. The sample was stirred and irradiated with a blue-LED light (LED-Illuminator, Luzchem, Gluocester, Canada), with emission at 450–460 nm and an output power of 100 mW/cm^2^. The Soret band absorbance was followed for the light irradiation, and its decay was followed through UV-vis absorption (350–800 nm).

### 4.6. Influence of pH for the Nanoparticles Behaviour and Stability

#### 4.6.1. pH Buffers

Aqueous buffer solutions prepared for the pH analysis were prepared using appropriated acid–base pairs, at the same molarity. Namely, glycine–HCl 0.01 M was used for buffers at pH 2.0, 2.6, 3.0 and 3.6. Sodium acetate–acetic acid 0.01 M was used for buffers at pH 4.0, 4.6, 5.0 and 5.6. Sodium phosphate–sodium biphosphate 0.01 M was used for buffers at pH 6.0, 6.6, 7.0, 7.6 and 8.0. Glycine–NaOH 0.01 M was used for buffers at pH 8.6, 9.0, 9.6, 10.0 and 10.6. The pH of all buffers was verified and adjusted, using a pH meter (Mettler Toledo, Columbus, OH, USA).

The influence of pH was analyzed through UV-vis absorption, measured between 350 and 800 nm. Free porphyrins were dissolved in DMF at a concentration of 1 mM and then diluted to 5 µM in the buffer for analysis, resulting in a final concentration of 5% DMF *v*/*v*. Nanoparticles suspensions were diluted in the buffer to analyze at a final concentration of 5 µM. The Soret band absorption and maximum wavelength were followed up, and the values were normalized according to the values obtained at pH 7.

#### 4.6.2. pH Driven Release

Porphyrin-loaded nanoparticles were suspended either in buffer glycine–HCl 0.01 M pH 3 or phosphate buffer 0.01 M pH 7, at a final concentration of 20 µM. The nanoparticles suspensions were stirred on an orbital shaker, at 200 rpm. For analysis, 500 µL of the nanoparticles suspension were taken and immediately centrifuged (10,000× *g*, 10 min). Afterwards, 200 µL of the supernatant were carefully taken and stored at 4 °C until analysis. For analysis, the samples were diluted with 1.8 mL of methanol, and excited at the porphyrin’s wavelength of the Soret’s band in methanol, for monitoring at the maximum emission wavelength, according to Table 8.

### 4.7. Photodynamic Antimicrobial Chemotherapy

All experiments used planktonic bacteria in the middle of the exponential phase of growth. For this, an aliquot of frozen bacteria was inoculated in 5 mL of culture media and incubated overnight, at 37 °C and constant stirring at 100 rpm. The optical density at 600 nm (OD_600_) was measured for the resulting culture, and further diluted at an OD_600_ = 0.1 in 5 mL of fresh culture media. This subculture was incubated for two hours, at 37 °C and constant stirring. Bacteria were collected, centrifuged (4000× *g*, 10 min), washed with sterile PB pH 7 and resuspended in 1 mL of PB pH 7, resulting in a bacterial suspension with around 10^8^ CFU/mL.

Free porphyrins were dissolved in DMSO, for a stock concentration of 1 mM. Then, the DMSO solution was geometrically diluted in DMSO, obtaining solutions ranging from 1000 to 7.8125 µM. Then, each dilution was diluted 1:10 in PB pH 7, resulting in a solution with 10% DMSO and concentrations of porphyrins ranging between 100 and 0.78125 µM. Porphyrin-loaded nanoparticles were diluted in PB pH 7, at a stock concentration of 100 µM and geometrically diluted until 0.78125 µM.

For the bacteriostatic effect analysis, bacteria were diluted to ~10^5^ CFU/mL in PB pH 7. Bacteria aliquots (50 µL) were delivered in a 96-well plate (Corning, Corning, NY, USA) and mixed with 50 µL of the treatment solution, for a final range of concentrations of 50 to 0.35625 µM. Bacteria and the treatment were incubated in the dark for 30 min. Afterwards, each well was irradiated with a vertical array of blue-LED light (LED-Illumintor, Luzchem, Gluocester, Canada) at 1.4 cm from the plate and with an intensity of 500 mW/cm^2^ for 30 s, resulting in a light dosage of 15 J/cm^2^. Light irradiance was routinely verified using a handheld powermeter (Lasercheck, Coherent, Santa Clara, CA, USA), centered at 455 nm. During the irradiation of each well, the rest of the plaque was protected from light with a black cardboard mold. A similar plaque was prepared, kept in the dark during the irradiation and worked as dark control. Without further incubation, 100 µL of culture media were added into each well and the initial optical density at 595 nm (OD_595_) was measured on a multiplate reader (iMark, Bio-Rad, Marnes-la-Coquette, France) as time-zero (OD_595_^0^). Each plate was incubated in the dark, at 37 °C, and its optical density was measured again (OD_595_^16^). Appropriated controls were prepared: a sample without bacteria and without treatment was used as a blank (G_B_), while a bacterial sample with PB pH 7 or PB pH 7 5% DMSO was used as growth control (G_C_). Growth was obtained as the difference between OD_595_^16^ and OD_595_^0^, while the normalized growth (G_N_) for each sample was obtained according to the Equation (3), where G_S_ is the growth of the sample. The MIC was considered to be the lowest concentration where the normalized growth was below 0.1.

(3) $$G_{N}=\frac{G_{S}-G_{B}}{G_{C}-G_{B}}$$

For the bactericidal effect analysis, bacteria were diluted to ~10^7^ CFU/mL in PB pH 7. Bacteria aliquots (50 µL) were delivered in a 96-well plate (Corning, Corning, NY, USA) and mixed with 50 µL of the treatment solution, descending from the MIC concentration values. Bacteria and the treatment were incubated in the dark for 30 min. Afterwards, each well was irradiated with a vertical array of blue-LED light (LED-Illumintor, Luzchem, Gluocester, Canada) at 1.4 cm from the plate and with an intensity of 500 mW/cm^2^ for 30 s, resulting in a light dosage of 15 J/cm^2^. Light irradiance was routinely verified using a handheld powermeter (Lasercheck, Coherent, Santa Clara, CA, USA), centered at 455 nm. During the irradiation of each well, the rest of the plaque was protected from light with a black cardboard mold. A similar plaque was prepared and kept in the dark during the irradiation and worked as a dark control. A bacterial control with PB pH 7 or PB pH 7 5% DMSO was used as growth control (G_C_). Without further incubation, bacterial suspensions were recovered and serially diluted in saline solution. Afterwards, 50 µL of the diluted bacterial suspension were plated into TSA culture media, using an automatic plater (EasySpyral, Interscience, Mourjou, France). Colony-forming units were counted using a colony counter (Scan100, Interscience, Mourjou, France). Bacterial survival was expressed as the logarithm of the concentration (log(CFU/mL)). The MBC was considered to be the lowest concentration where a reduction of 3-log was observed.

### 4.8. Statistical Analysis

Physical and photophysical experiments were performed at least in duplicate. Microbiological experiments were performed at least in triplicate. The obtained DLS raw data were analyzed with GraphPad Prism 6.01, and data were fit to a Gaussian model, excluding the values with less than 1% of presence. The obtained data were validated through the analysis of their R square coefficient and through the analysis of the residuals with a D’Agostino–Pearson Omnibus K2 test. Statistical significance was determined using a Two-Way ANOVA and a Tukey’s test for multiple comparisons, with 95% of the cohort.

Graphs presented throughout this work were prepared with RStudio (R version 4.0.4), using ggplot and diplyr libraries.

## Figures and Tables

**Table 1:** Physical characteristics of nanoparticles.

Nanoparticles	DLS				PDI	Zeta Potential (mV) ^b^
	Mean Size (nm)	Range (D_95_) ^a^	R^2^ Gaussian Model Fitting	Normality of Residuals (*p* Value)		
@AcLi	184.3	57.16–311.44	0.7517	0.2232	0.183 ± 0.026	−22.1 ± 2.041
THPP@AcLi	160.4	51.29–262.98	0.8112	0.4243	0.126 ± 0.028	−20.8 ± 0.474
T(OAc)PP@AcLi	199.6	78.92–320.28	0.8535	0.1327	0.122 ± 0.117	−21.180 ± 0.887
T(MAP)PP@AcLi	886.2	597.8–1174.6	0.8931	0.8561	0.653 ± 0.145	−2.808 ± 1.461
T(PrOH)PyP@AcLi	1348	880.6–1815.4	0.6612	0.9621	0.457 ± 0.016	−9.962 ± 1.301
ZnTHPP@AcLi	208.2	77.88–338.52	0.8721	0.2714	0.117 ± 0.020	−24.140 ± 1.618

^a^ The range denotes the distribution of 95% of the nanoparticles in suspension, calculated as ±2σ obtained from the Gaussian fitting. ^b^ Zeta potential measured in phosphate buffer 0.01 M, pH 7.0.

**Table 2:** Absorption spectra properties at the Soret band wavelength of different porphyrins dissolved in DMF or PB pH 7, or encapsulated in acetylated lignin nanoparticles, and suspended in PB pH 7. The effect of encapsulation on the absorption capacity of the porphyrins is described as the ratio of absorption coefficients of the encapsulated porphyrins and of the free porphyrins (ε/ε_Free_). Additionally, the shift of the observed Soret bands wavelength upon encapsulation is given (Δ_λ_).

Porphyrin	Free in DMF		Free in PB pH 7 ^a^		Free in PB pH 7 vs. Free in DMF		Encapsulated in PB pH 7		Encapsulated vs. Free in DMF		Encapsulated vs. Free in PB	
	ε (M^−1^ cm^−1^)	λ (nm)	ε (M^−1^ cm^−1^)	λ (nm)	ε_PB_/ε_DMF_	Δ_λ_ (nm)	ε (M^−1^ cm^−1^)	λ (nm)	ε/ε_Free_	Δ_λ_ (nm)	ε/ε_Free_	Δ_λ_ (nm)
THPP	35.0668 × 10^4^	424	5.9361 × 10^4^	440	0.169	16	12.9984 × 10^4^	434	0.3707	10	2.1897	−6
T(OAc)PP	36.1354 × 10^4^	419	4.4123 × 10^4^	429	0.122	10	12.0609 × 10^4^	426	0.3338	7	2.7335	−3
T(MAP)PP	20.1871 × 10^4^	422	15.7138 × 10^4^	425	0.778	3	20.7684 × 10^4^	432	1.0288	10	1.3217	−7
T(PrOH)PyP	13.2376 × 10^4^	427	13.2908 × 10^4^	419	1.004	−8	1.3472 × 10^4^	448	0.1018	21	0.1014	−19
ZnTHPP	47.7749 × 10^4^	430	5.9163 × 10^4^	426	0.124	−4	18.5219 × 10^4^	435	0.3877	5	3.1307	−9

^a^ Free porphyrins in PB pH = 7, 5% DMF.

**Table 3:** Fluorescent quantum yields for porphyrins dissolved in DMF, in PB pH 7 0.5% DMF or encapsulated in acetylated lignin nanoparticles and suspended in PB pH 7. Fluorescent emission was measured at a porphyrin concentration of 0.5 µM, upon excitation at 425 nm, at 25 °C, and using TPP as a standard (Φ_F_ = 0.11 in toluene).

Porphyrin	Fluorescent Quantum Yield (Φ_F_)			Φ_F encapsulated/_Φ_F free in PB_ (PB pH 7)
	Free Porphyrin (DMF)	Free Porphyrin (PB pH 7)	Encapsulated Porphyrin (PB pH 7)	
THPP	0.1696	0.0034	0.0103	3.029
T(OAc)PP	0.1176	0.0089	0.0690	7.753
T(MAP)PP	0.1341	0.1349	0.0310	0.230
T(PrOH)PyP ^a^	0.1933	0.4219	3.5835 ^a^	8.494 ^a^
ZnTHPP	0.0648	0.0063	0.0101	1.603

^a^ The obtained value of the T(PrOH)PyP@AcLi fluorescence is a result of low fluorescence and low absorbance obtained after the light scattering correction, resulting in an extremely high value that is unlikely to have any relevance.

**Table 4:** Singlet oxygen quantum yield for the free porphyrins, calculated through the decay of singlet oxygen phosphorescence at 1270 nm.

Porphyrin	Singlet Oxygen Quantum (Φ_Δ_)		
	Free Porphyrin ^a^	Literature ^b^	
THPP	0.5900	0.57 (DMA oxidation, DMF, air, λ_Ex_ = 401 nm)	[37]
T(OAc)PP	0.6560		
T(MAP)PP	0.6321	0.51 (DMA oxidation, DMF, air, λ_Ex_ = 420 nm)	[38]
T(PrOH)PyP	0.5804		
ZnTHPP	0.7320		

^a^ Porphyrins dissolved in DMF, with an optical density of around 0.18 at the excitation wavelength (355 nm), using phenalenone as standard (Φ_Δ_ = 1 [36]). ^b^ The reported values found in the literature are presented with the method used, the solvent, the kind of aeration and the wavelength of excitation.

**Table 5:** Minimal inhibitory concentrations found for *S. aureus* and *E. coli*, when treated with the free or encapsulated porphyrins. Irradiated bacteria were incubated with the treatment for 30 min, before irradiation under blue-LED light (15 J/cm^2^).

Porphyrin	*S. aureus*		*E. coli*	
	Light	Dark	Light	Dark
THPP	0.78 µM	>50 µM	>50 µM	>50 µM
THPP@AcLi	>25 µM	>50 µM	>50 µM	>50 µM
T(OAc)PP	>50 µM	>50 µM	>50 µM	>50 µM
T(OAc)PP@AcLi	>50 µM	>50 µM	>50 µM	>50 µM
T(MAP)PP	1.56 µM	3.13 µM	1.56 µM	>50 µM
T(MAP)PP@AcLi	>50 µM	>50 µM	50 µM	50 µM
T(PrOH)PyP	6.25 µM	50 µM	1.56 µM	>50 µM
T(PrOH)PyP@AcLi	>50 µM	>50 µM	50 µM	50 µM
ZnTHPP	0.78 µM	3.13 µM	>50 µM	>50 µM
ZnTHPP@AcLi	50 µM	50 µM	50 µM	50 µM

**Table 6:** Minimal bactericidal concentrations found for *S. aureus* and *E. coli*, when treated with the free or encapsulated porphyrins. Irradiated bacteria were incubated with the treatment for 30 min, before irradiation under blue-LED light (15 J/cm^2^). In parenthesis is indicated the rate MIC/MBC for the concentrations where MBC could be precisely determined.

Porphyrin	*S. aureus*		*E. coli*	
	Light	Dark	Light	Dark
THPP	0.0488 µM (16)	0.1953 µM	>50 µM	>50 µM
THPP@AcLi	0.7813 µM (32)	>1.5625 µM	>50 µM	>50 µM
T(OAc)PP	>50 µM	>50 µM	>50 µM	>50 µM
T(OAc)PP@AcLi	>50 µM	>50 µM	>50 µM	>50 µM
T(MAP)PP	0.0500 µM (32)	>0.2 µM	0.4 µM (4)	>0.4 µM
T(MAP)PP@AcLi	>50 µM	>50 µM	>50 µM	>50 µM
T(PrOH)PyP	0.200 µM (32)	>0.2 µM	0.2 µM (8)	>0.2 µM
T(PrOH)PyP@AcLi	1.0 µM (50)	>2 µM	6.25 µM (8)	>50 µM
ZnTHPP	0.0977 µM (8)	>0.1953 µM	>50 µM	>50 µM
ZnTHPP@AcLi	6.25 µM (8)	>50 µM	>50 µM	>50 µM

**Table 7:** Solvents used for the starting solution of each type of nanoparticle analyzed in the present work.

Porphyrin	Solvent or Solvents Mixtures
THPP	Acetone
T(OAc)PP	Acetone:DMF 9:1
T(MAP)PP	Acetone:DMSO 9:1
T(PrOH)PyP	Acetone:DMSO 9:1
ZnTHPP	THF

**Table 8:** Wavelengths for excitation and emission, for the porphyrins dissolved in methanol with 10% aqueous buffer.

Porphyrin	Excitation Wavelength	Emission Wavelength
THPP	419 nm	652 nm
T(OAc)PP	415 nm	648 nm
T(MAP)PP	418 nm	652 nm
T(PrOH)PyP	427 nm	650 nm
ZnTHPP	424 nm	607 nm

## Data Availability

No datasets were generated.

## Supplementary Materials

The following are available online at https://www.mdpi.com/article/10.3390/antibiotics10050513/s1. Figure S1. Comparison of the spectra of (A) THPP, (B) T(MAP)PP, (C) T(OAc)PP, (D) T(PrOH)PyP and (E) ZnTHPP as free porphyrins (black), or as the encapsulated porphyrins before (blue) and after (red) the correction of the baseline. Figure S2. Spectra of (A) THPP, (B) T(OAc)PP, (C) T(MAP)PP, (D) T(PrOH)PyP, and (E) ZnTHPP upon water addition. Porphyrins were initially dissolved in (A) acetone, (B) acetone:DMF 9:1, (C) acetone:DMSO 9:1, (D) acetone:DMSO 9:1, and (E) THF, at a concentration of 5 µM (black line), through increasing water addition. Figure S3. Normalized absorbance (solid lines) and emission (dashed lines) spectra of (A) THPP, (B) T(MAP)PP, (C) T(OAc)PP, (D) T(PrOH)PyP and (E) ZnTHPP, dissolved in DMF (black), in PB pH 7, 0.5% DMF (blue), or as encapsulated porphyrins (red). Emission spectra were collected from a solution at 0.5 µM, at 25 ºC, with excitation at 425 nm. Figure S4. Fluorescence emission (black solid lines) and excitation (black and blue dashed lines) spectra of the free porphyrins in DMF (A, B, C, D, E) or in aqueous buffer at pH 2 (F, G, H, I, J); or as encapsulated porphyrins suspended in pH 7 (K, L, M, N, O) or in pH 2 (P, Q, R, S, T). The black horizontal line indicates the wavelength of the Soret band of each porphyrin in DMF, while the dashed horizontal lines indicate the peak of the emission bands of each porphyrin in DMF, also indicating as well the emission wavelength monitored in the excitation spectra. Emission spectra were after excitation at 425 nm; all spectra were measured at 25 °C, from a solution 0.5 µM of the corresponding porphyrin. Figure S5. Slopes of the decay of singlet oxygen phosphorescence, detected at 1270 nm, measured through near infrared spectroscopy, as a function of laser energy. Porphyrins were dissolved in DMF, with an absorption of around 0.18 at 355 nm, the excitation wavelength. Phenalenone in DMF was used as a standard (ΦΔ = 1). Figure S6. Absorption spectra of free porphyrins (A) THPP, (B) T(OAc)PP, (C) T(MAP)PP, (D) T(PrOH)PyP and (E) ZnTHPP, after irradiation under blue LED light (100 mW/cm^2^). Porphyrins were dissolved in DMF, 5 µM, with constant stirring on an open quartz cell. Shown spectra are the average of two individual experiments. Figure S7. Absorption spectra of encapsulated porphyrins (A) **THPP@AcLi**, (B) T(OAc)PP@AcLi, (C) T(MAP)PP@AcLi, (D) T(PrOH)PyP@AcLi and (E) ZnTHPP@AcLi, after irradiation under blue-LED light (100 mW/cm^2^). Nanoparticles were suspended in PB pH 7, at 5 µM of their corresponding porphyrin, with constant stirring on an open quartz cell. Shown spectra are the average of two individual experiments. Figure S8. Comparison of spectra for T(PrOH)PyP before light irradiation (black), after light irradiation (blue), and the raw porphyrin, THPyP in DMF (red). Figure S9. Effect of pH on the UV-vis absorbance profile of the free porphyrins (A) THPP, (C) T(OAc)PP, (E) T(MAP)PP, (G) T(PrOH)PyP, (I) ZnTHPP, or as encapsulated porphyrins (B) THPP@AcLi, (D) T(OAc)PP@AcLi, (F) T(MAP)PP@AcLi, (H) T(PrOH)PyP@AcLi, (J) ZnTHPP@AcLi. Red lines represent the pH below 7, while blue lines represent the pH above 7. Both free and encapsulated porphyrins were set in a final concentration of 5 µM of their corresponding porphyrin, in an adequate aqueous buffer. Free porphyrins were dissolved in DMF and diluted into an aqueous buffer, for a final DMF concentration of 5%. Table S1. Free porphyrin observed at initial and final observation times, expressed as percent of free porphyrin, with respect to the global amount of encapsulated porphyrins (20 µM). Similar superscript letters indicate the pairs that have differences with a statistical significance, found as *p* < 0.05 with a two-way ANOVA, and a multiple comparisons Tukey’s test. Figure S10. Growth inhibition of Staphylococcus aureus, after treatment with (A) THPP, (B) THPP@AcLi, (C) T(OAc)PP, (D) T(Oac)PP@AcLi, I T(MAP)PP, (F) T(MAP)PP@AcLi, (G) T(PrOH)PyP, (H) T(PrOH)PyP@AcLi, (I) ZnTHPP and (J) ZnTHPP@AcLi. Bacteria were incubated for 30 min and then irradiated with blue-LED light (15 J/cm^2^, white symbols) or incubated in the dark (grey symbols). Afterwards, culture medium was added and bacteria were incubated at 37 °C, in the dark for 16 h. Results shown are the average of three independent experiments. Figure S11. Growth inhibition of Escherichia coli, after treatment with (A) THPP, (B) THPP@AcLi, (C) t(OAc)PP, (D) t(OAc)PP@AcLI, (E) T(MAP)PP, (F) T(MAP)PP@AcLi, (G) T(PrOH)PyP, (H) T(PrOH)PyP@AcLi, (I) ZnTHPP and (J) ZnTHPP@AcLi. Bacteria were incubated for 30 min and then irradiated with blue-LED light (15 J/cm^2^, white symbols) or incubated in the dark (grey symbols). Afterwards, culture medium was added and bacteria were incubated at 37 °C, in the dark for 16 h. Results shown are the average of three independent experiments. Figure S12. Bacterial survival of S. aureus, after treatment with (A) THPP, (B) THPP@AcLi, (c) T(OAc)PP, (d) T(OAc)PP@Ii, (E) T(MAP)PP, (F) T(MAP)PP@AcLi, (G) T(PrOH)PyP, (H) T(PrOH)PyP@AcLi, (I) ZnTHPP and (J) ZnTHPP@AcLi. Bacteria were incubated for 30 min and then irradiated with blue-LED light (15 J/cm^2^, white symbols) or incubated in the dark (grey symbols). Afterwards, bacteria were diluted and plated in Petri dishes, which were incubated in the dark 16 h, before counting colonies. The dotted line indicates the limit of quantification (2.9 log), while the dashed line indicates a diminish of at least 3 log. Figure S13. Bacterial survival of *E. coli*, after treatment with (A) THPP, (B) THPP@AcLi, (C) T(OAc)PP, (D) T(OAc)IAcLi, (E) T(MAP)PP, (F) T(MAP)PP@AcLi, (G) T(PrOH)PyP, (H) T(PrOH)PyP@AcLi, (I) ZnTHPP and (J) ZnTHPP@AcLi. Bacteria were incubated for 30 min and then irradiated with blue-LED light (15 J/cm^2^, white symbols) or incubated in the dark (grey symbols). Afterwards, bacteria were diluted and plated in Petri dishes, which were incubated in the dark for 16 h, before counting colonies. The dotted line indicates the limit of quantification (2.9 log), while the dashed line indicates a decrease of at least 3 log.
