# Supplementary material for: Photophysical and Antibacterial Properties of Porphyrins Encapsulated inside Acetylated Lignin Nanoparticles
Source: Antibiotics (Basel). 2021 Apr 30;10(5):513. doi: 10.3390/antibiotics10050513 (PMC8147155; doi:10.3390/antibiotics10050513)
Supplement: Supplementary file 1 [file antibiotics-10-00513-s001.zip › antibiotics-1186517-supplementary.pdf]

Supplementary Data

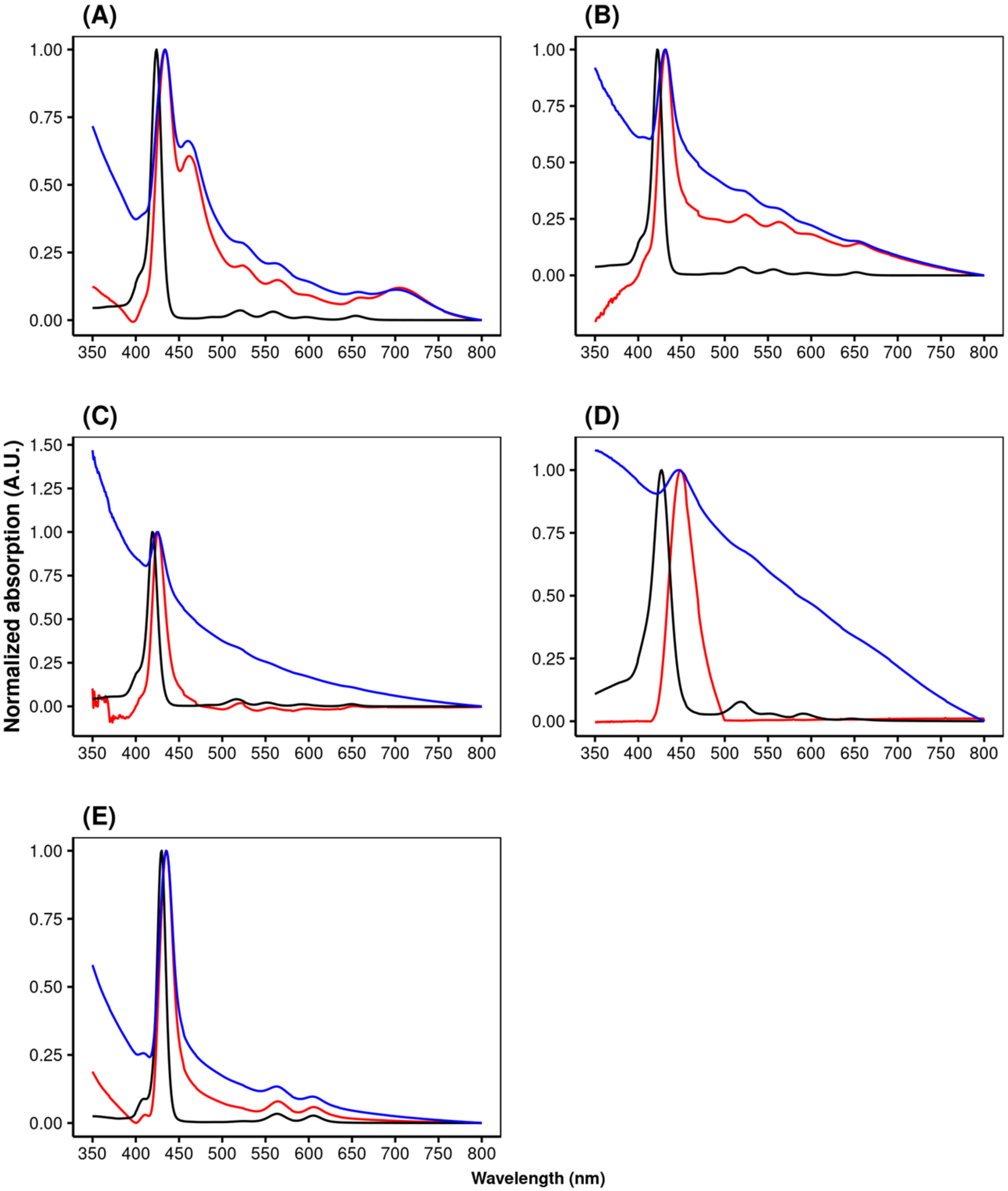

Supplementary Figure S1. Comparison of the spectra of (A) **THPP**, (B) **T(MAP)PP**, (C) **T(OAc)PP**, (D) **T(PrOH)PyP** and (E) **ZnTHPP** as free porphyrins (black), or as the encapsulated porphyrins before (blue) and after (red) the correction of the baseline.

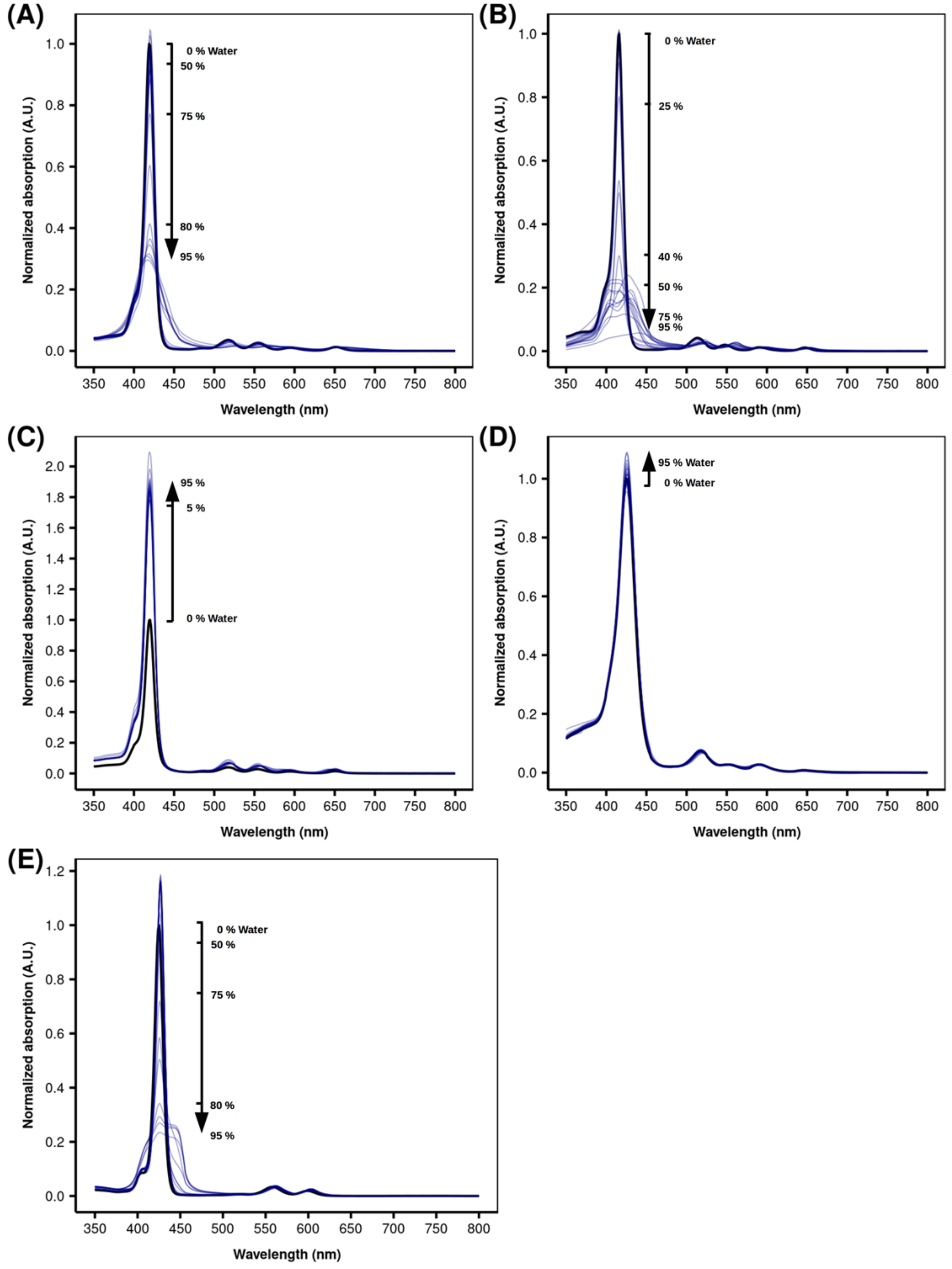

Supplementary Figure S2. Spectra of (A) **THPP**, (B) **T(OAc)PP**, (C) **T(MAP)PP**, (D) **T(PrOH)PyP**, and (E) **ZnTHPP** upon water addition. Porphyrins were initially dissolved in (A) acetone, (B) acetone:DMF 9:1, (C) acetone:DMSO 9:1, (D) acetone:DMSO 9:1, and (E) THF, at a concentration of 5 µM (black line), through increasing water addition.

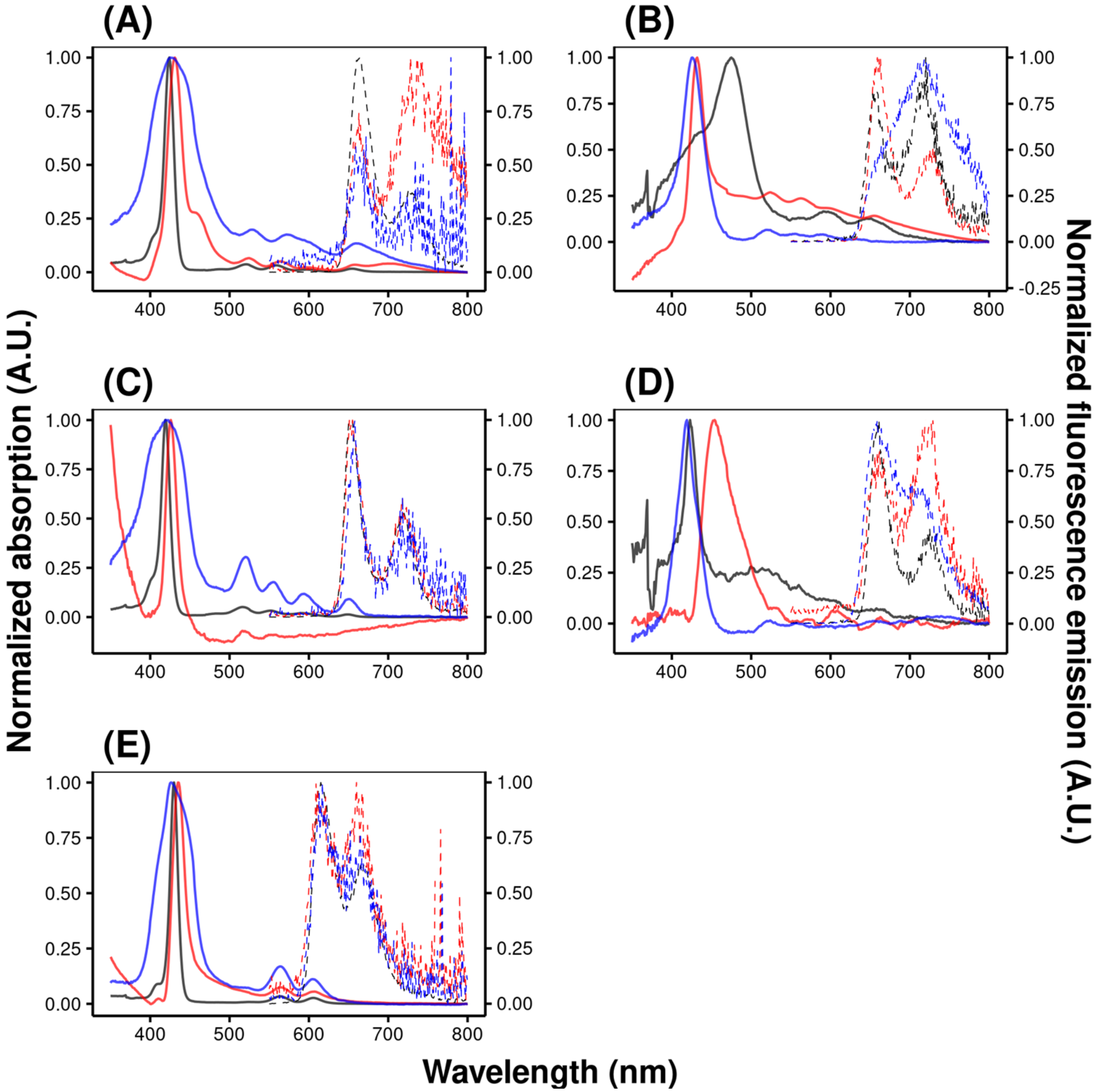

Supplementary Figure S3. Normalized absorbance (solid lines) and emission (dashed lines) spectra of (A) **THPP**, (B) **T(MAP)PP**, (C) **T(OAc)PP**, (D) **T(PrOH)PyP** and (E) **ZnTHPP**, dissolved in DMF (black), in PB pH 7, 0.5% DMF (blue), or as encapsulated porphyrins (red). Emission spectra were collected from a solution at 0.5 µM, at 25 ºC, with excitation at 425 nm.

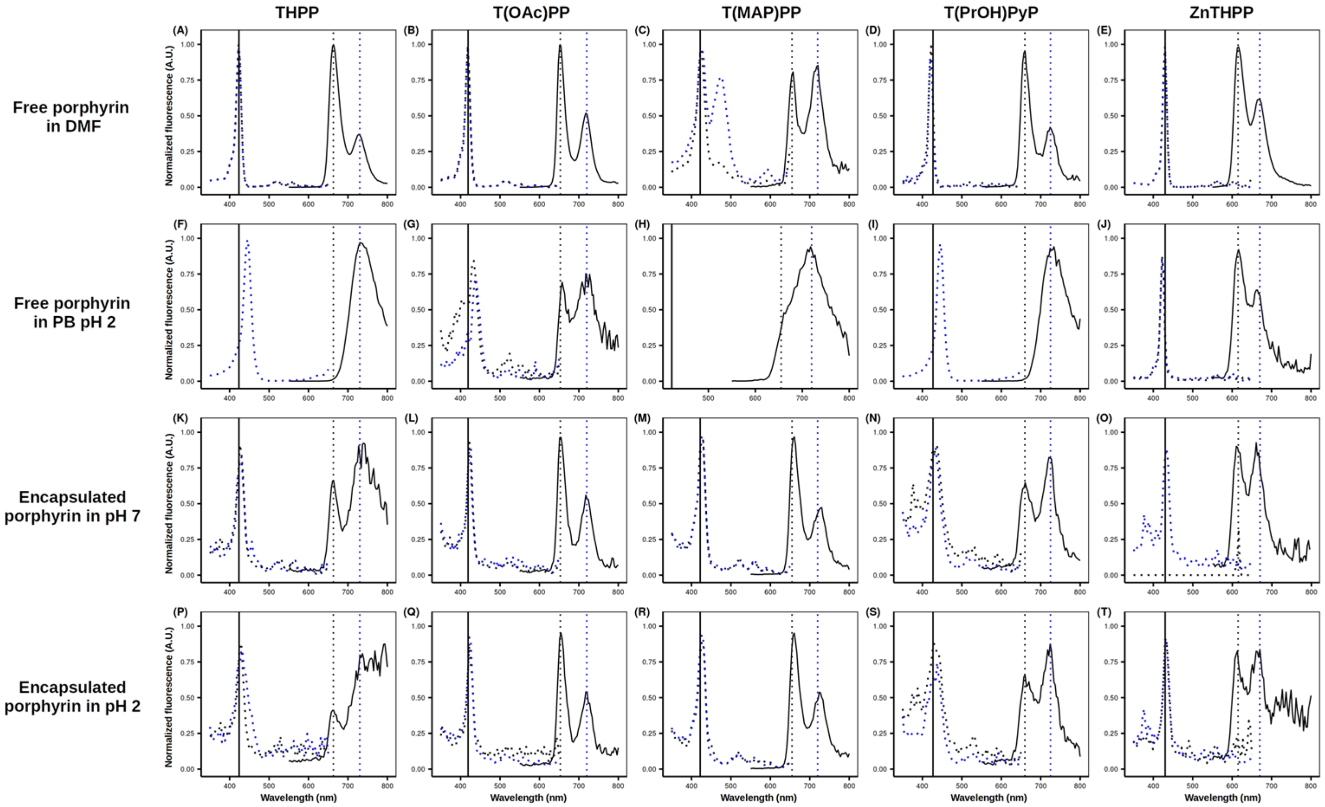

Supplementary Figure S4. Fluorescence emission (black solid lines) and excitation (black and blue dashed lines) spectra of the free porphyrins in DMF (A, B, C, D, E) or in aqueous buffer at pH 2 (F, G, H, I, J); or as encapsulated porphyrins suspended in pH 7 (K, L, M, N, O) or in pH 2 (P, Q, R, S, T). The black horizontal line indicates the wavelength of the Soret band of each porphyrin in DMF, while the dashed horizontal lines, indicate the peak of the emission bands of each porphyrin in DMF, indicating as well the emission wavelength monitored in the excitation spectra. Emission spectra were after excitation at 425 nm; all spectra were measured at 25 ºC, from a solution 0.5 µM of the corresponding porphyrin.

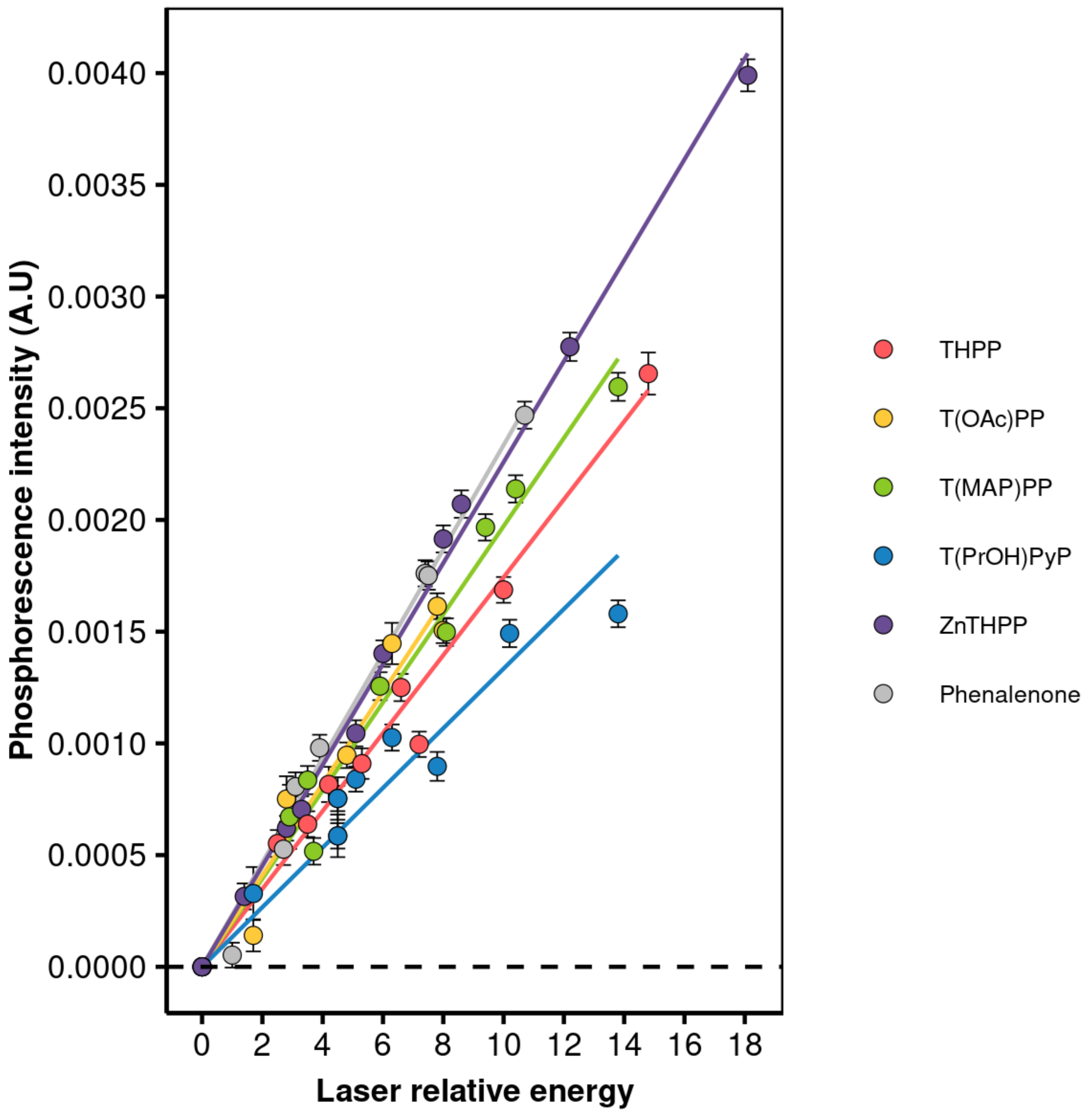

Supplementary Figure S5. Slopes of the decay of singlet oxygen phosphorescence, detected at 1270 nm, measured through near infrared spectroscopy, as a function of laser energy. Porphyrins were dissolved in DMF, with an absorption of around 0.18 at 355 nm, the excitation wavelength. Phenalenone in DMF was used as a standard ($\Phi_\Delta = 1$).

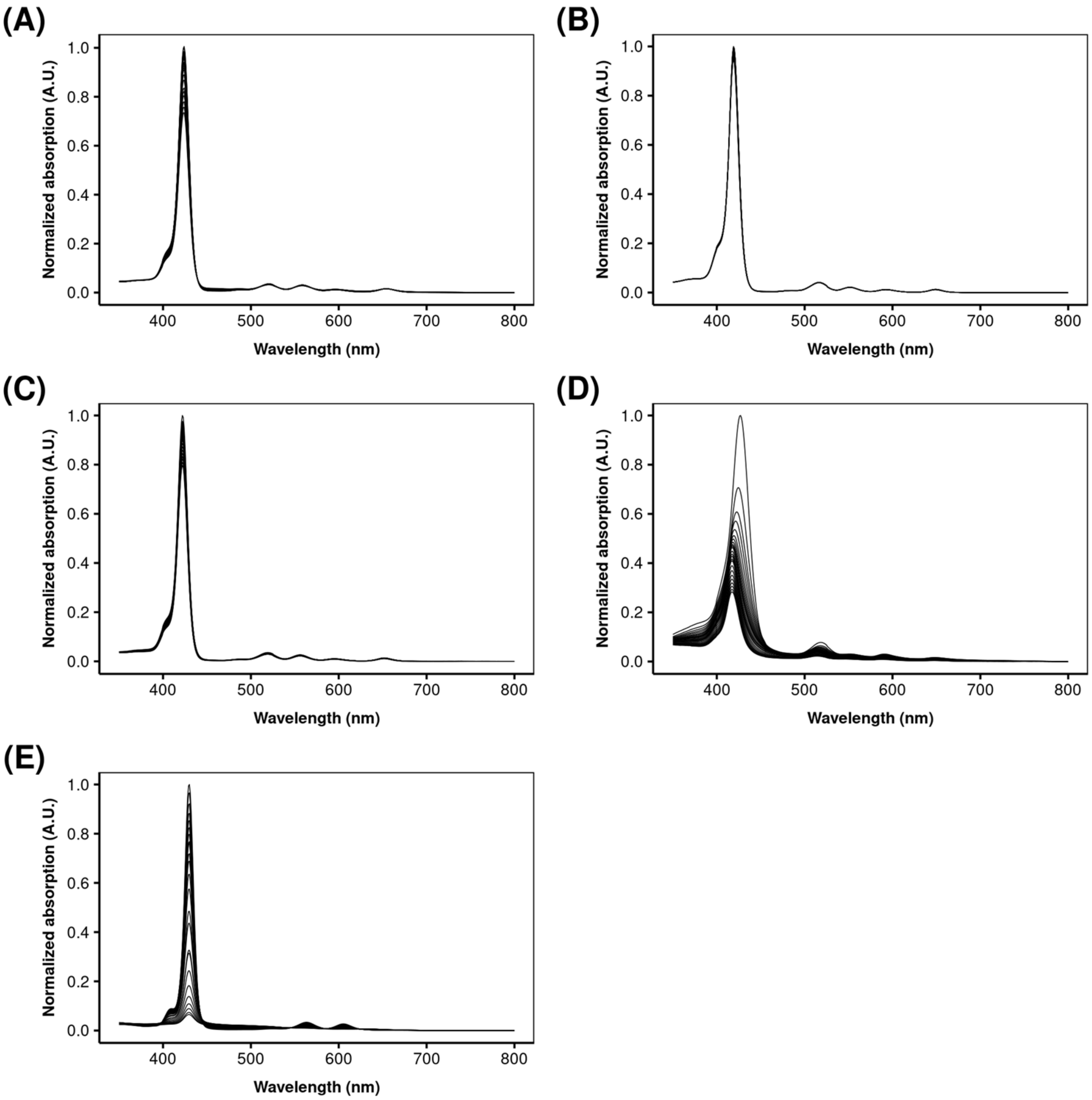

Supplementary Figure S6. Absorption spectra of free porphyrins (A) **THPP**, (B) **T(OAc)PP**, (C) **T(MAP)PP**, (D) **T(PrOH)PyP** and (E) **ZnTHPP**, after irradiation under blue LED light (100 mW/cm^2). Porphyrins were dissolved in DMF, 5 µM, with constant stirring on an open quartz cell. Shown spectra are the average of two individual experiments.

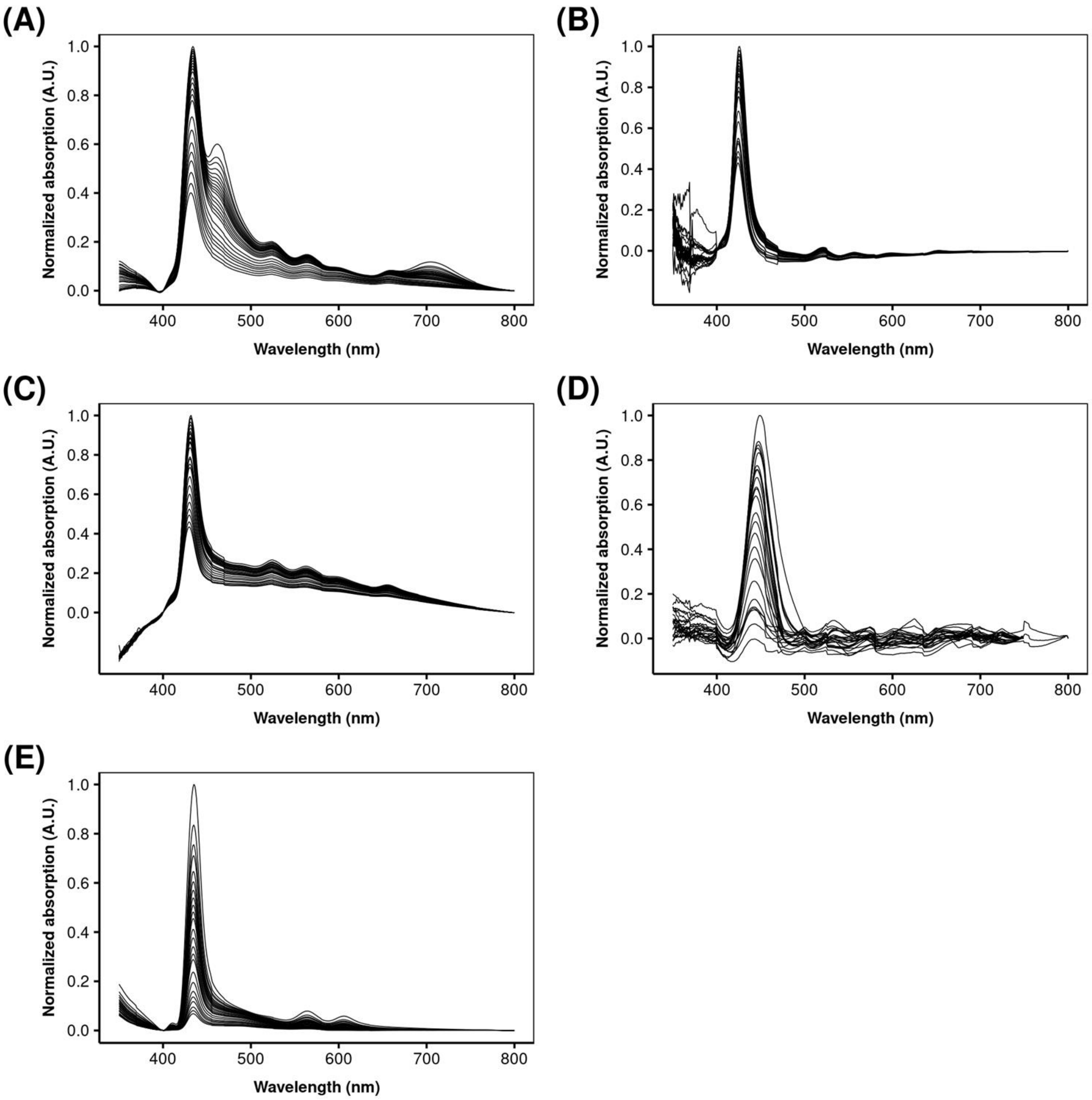

Supplementary Figure S7. Absorption spectra of encapsulated porphyrins (A) **THPP@AcLi**, (B) **T(OAc)PP@AcLi**, (C) **T(MAP)PP@AcLi**, (D) **T(PrOH)PyP@AcLi** and (E) **ZnTHPP@AcLi**, after irradiation under blue LED light (100 mW/cm^2). Nanoparticles were suspended in PB pH 7, at 5 μM of their corresponding porphyrin, with constant stirring on an open quartz cell. Shown spectra are the average of two individual experiments.

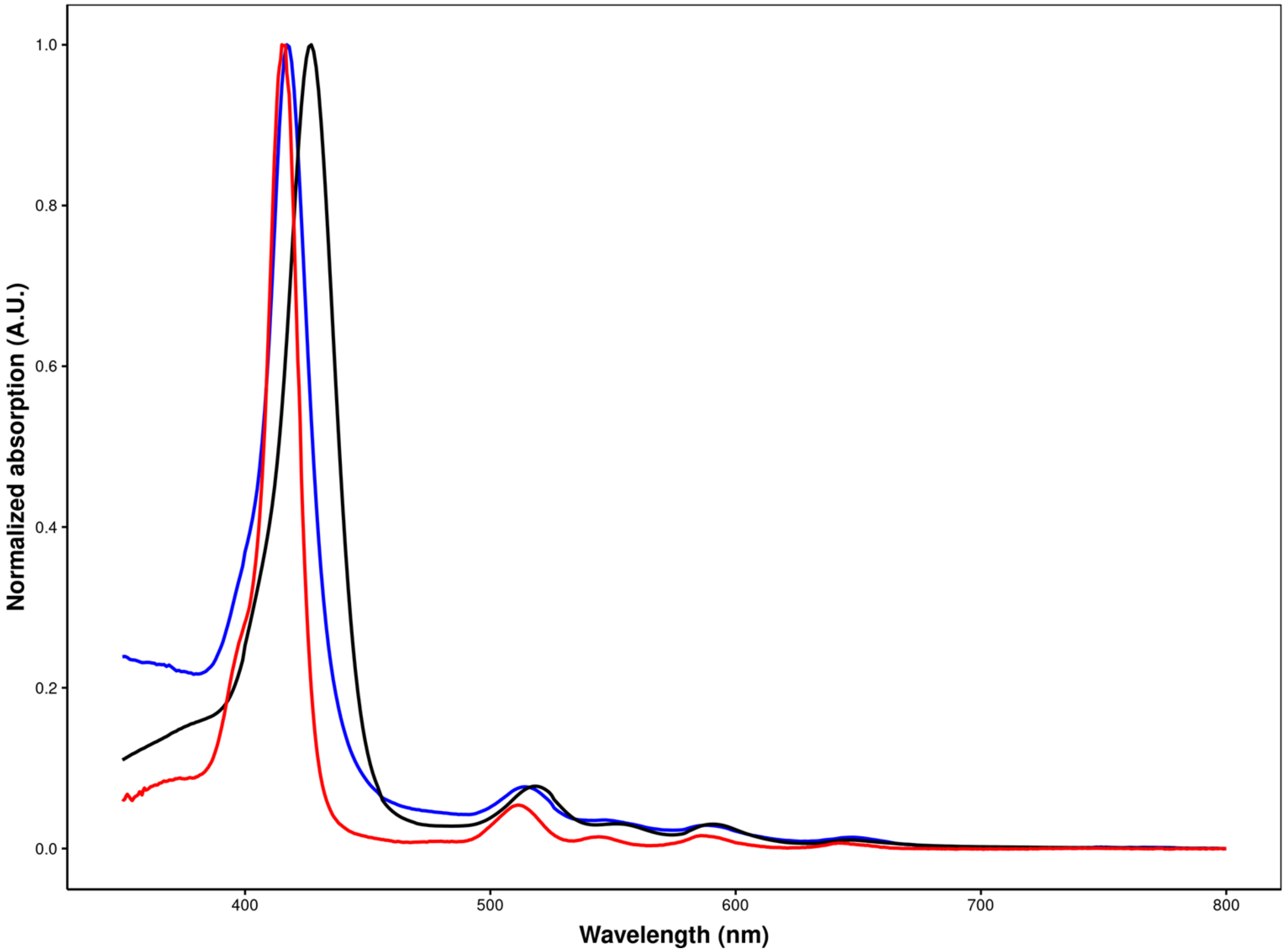

Supplementary Figure S8. Comparison of spectra for **T(PrOH)PyP** before light irradiation (black), after light irradiation (blue), and the raw porphyrin, THPyP in DMF (red).

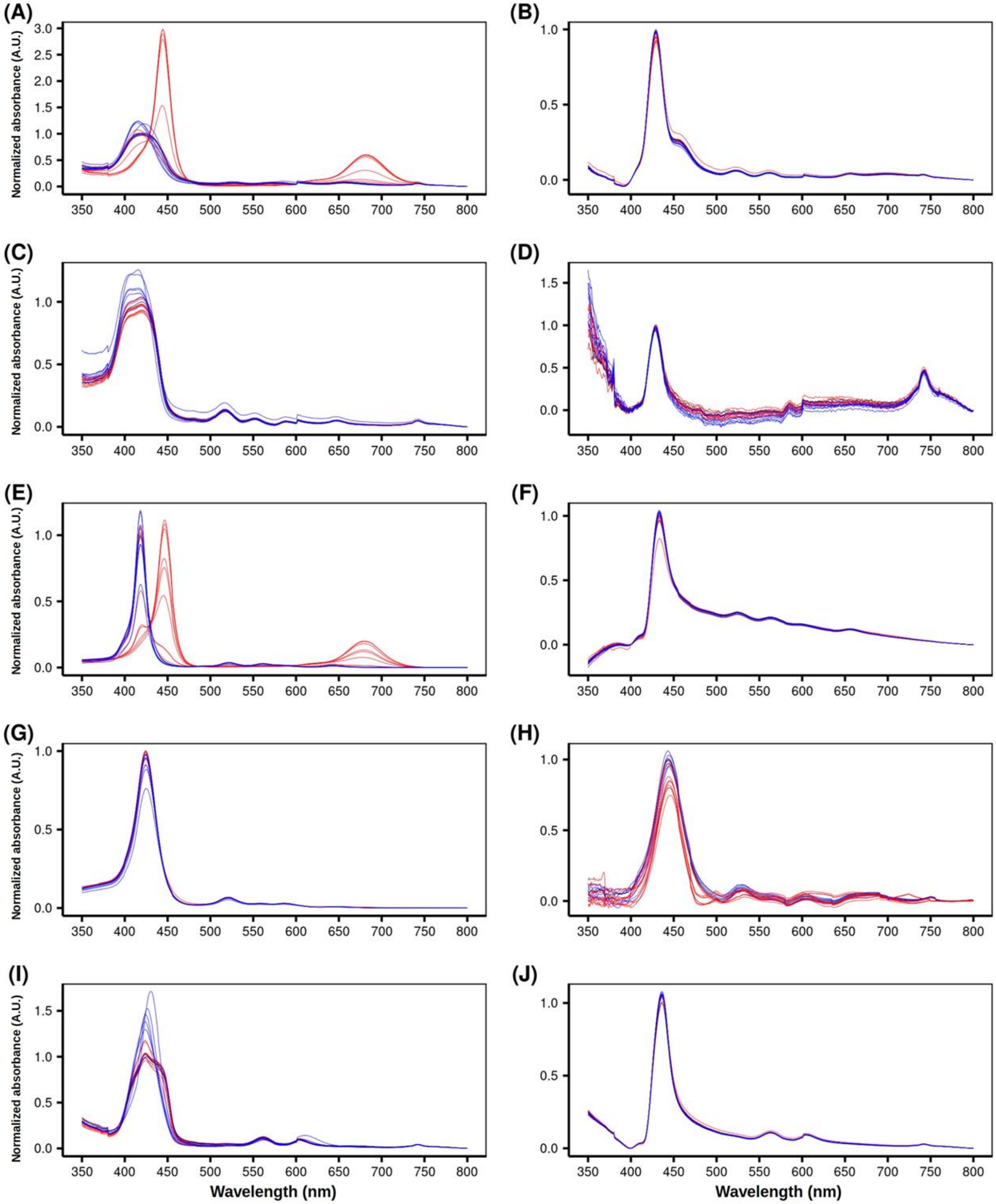

Supplementary Figure S9. Effect of pH on the UV-vis absorbance profile of the free porphyrins (A) **THPP**, (C) **T(OAc)PP**, (E) **T(MAP)PP**, (G) **T(PrOH)PyP**, (I) **ZnTHPP**, or as encapsulated porphyrins (B) **THPP@AcLi**, (D) **T(OAc)PP@AcLi**, (F) **T(MAP)PP@AcLi**, (H) **T(PrOH)PyP@AcLi**, (J) **ZnTHPP@AcLi**. Red lines represent the pH below 7, while blue lines represent the pH above 7. Both free and encapsulated porphyrins were set in a final concentration of 5 µM of their corresponding porphyrin, in an adequate aqueous buffer. Free porphyrins were dissolved in DMF and diluted into an aqueous buffer, for a final DMF concentration of 5%.

Supplementary Table S1. Free porphyrin observed at initial and final observation times, expressed as percent of free porphyrin, with respect to the global amount of encapsulated porphyrins (20 µM). Similar superscript letters indicate the pairs that have differences with a statistical significance, found as $P < 0.05$ with a Two-way ANOVA, and a multiple comparisons Tukey's test.

Porphyrin	pH 3		pH 7	
	0 hours	96 hours	0 hours	96 hours
THPP	1.67 ± 0.63^{a}	1.11 ± 0.17	7.23 ± 0.73^{a}	2.81 ± 0.16
T(OAc)PP	16.82 ± 0.02	7.58 ± 0.49	10.32 ± 5.30	9.00 ± 0.75
T(MAP)PP	9.38 ± 1.96^{b}	$16.4 \pm 0.55^{b,c}$	5.07 ± 4.94	0.97 ± 0.32^{c}
T(PrOH)PyP	3.25 ± 0.87	3.22 ± 0.17	3.46 ± 1.12	2.07 ± 0.21
ZnTHPP	1.76 ± 0.04^{d}	1.09 ± 0.50	$11.86 \pm 3.34^{d,e}$	2.56 ± 0.47^{e}

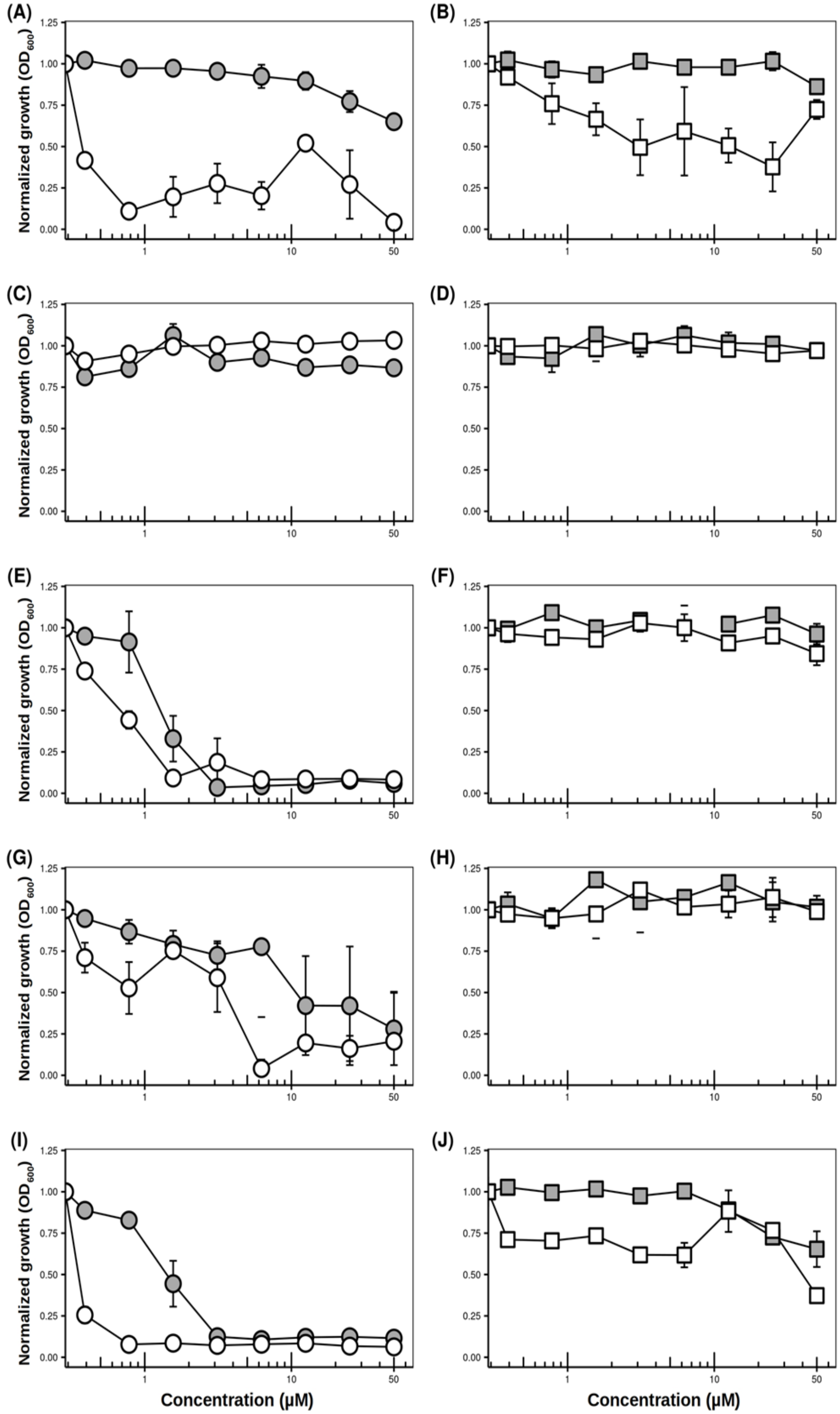

Supplementary Figure S10. Growth inhibition of *Staphylococcus aureus*, after treatment with (A) **THPP**, (B) **THPP@AcLi**, (C) **T(OAc)PP**, (D) **T(OAc)PP@AcLi**, (E) **T(MAP)PP**, (F) **T(MAP)PP@AcLi**, (G) **T(PrOH)PyP**, (H) **T(PrOH)PyP@AcLi**, (I) **ZnTHPP** and (J) **ZnTHPP@AcLi**. Bacteria were incubated for 30 minutes and then irradiated with blue-LED light (15 J/cm^2, white symbols) or incubated in the dark (grey symbols). Afterwards, culture medium was added and bacteria were incubated at 37 ººC, in the dark for 16 hours. Results shown are the average of three independent experiments.

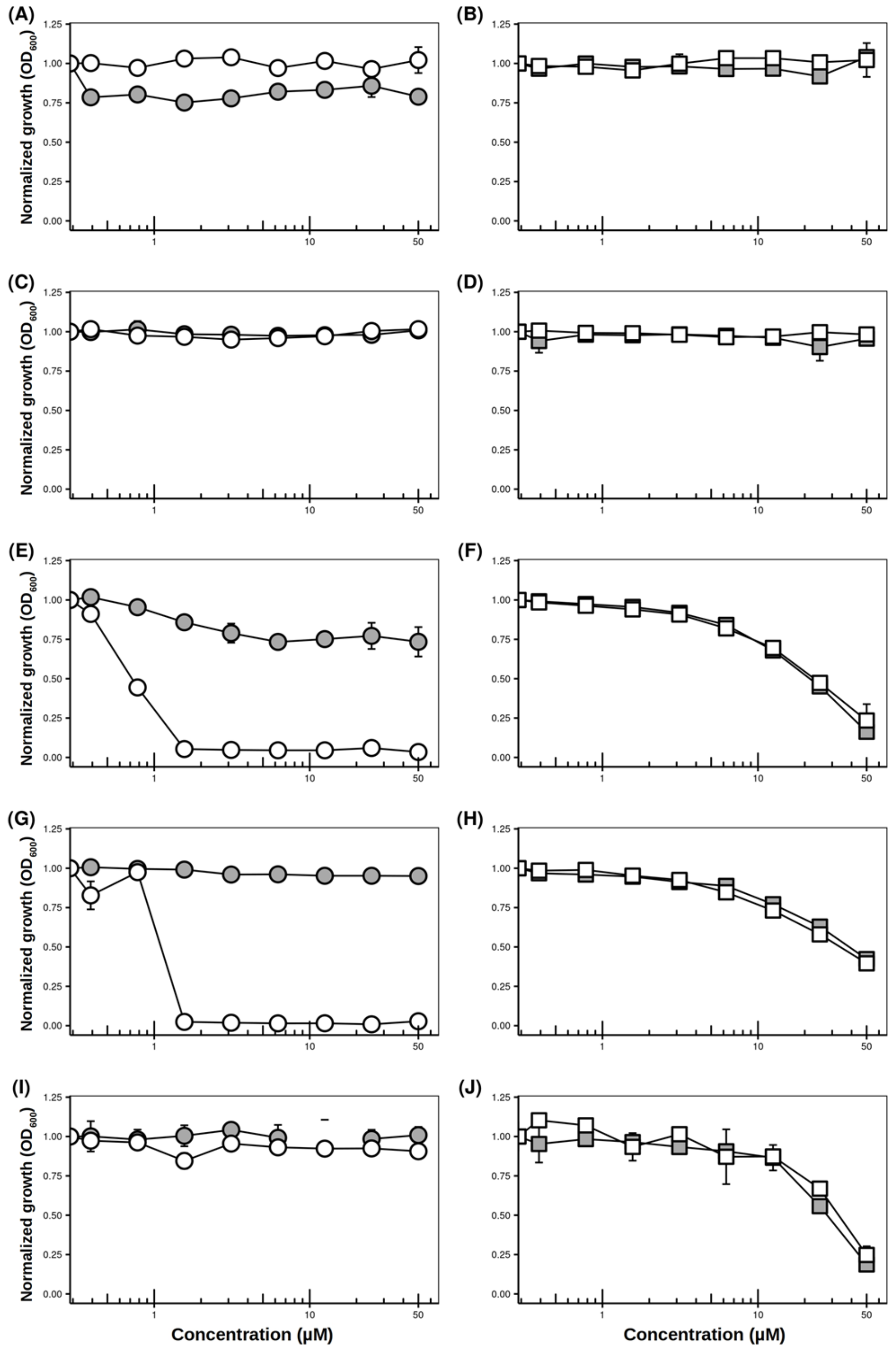

Supplementary Figure S11. Growth inhibition of *Escherichia coli*, after treatment with (A) **THPP**, (B) **THPP@AcLi**, (C) **T(OAc)PP**, (D) **T(OAc)PP@AcLi**, (E) **T(MAP)PP**, (F) **T(MAP)PP@AcLi**, (G) **T(PrOH)PyP**, (H) **T(PrOH)PyP@AcLi**, (I) **ZnTHPP** and (J) **ZnTHPP@AcLi**. Bacteria were incubated for 30 minutes and then irradiated with blue-LED light (15 J/cm^2, white symbols) or incubated in the dark (grey symbols). Afterwards, culture medium was added and bacteria were incubated at 37 ººC, in the dark for 16 hours. Results shown are the average of three independent experiments.

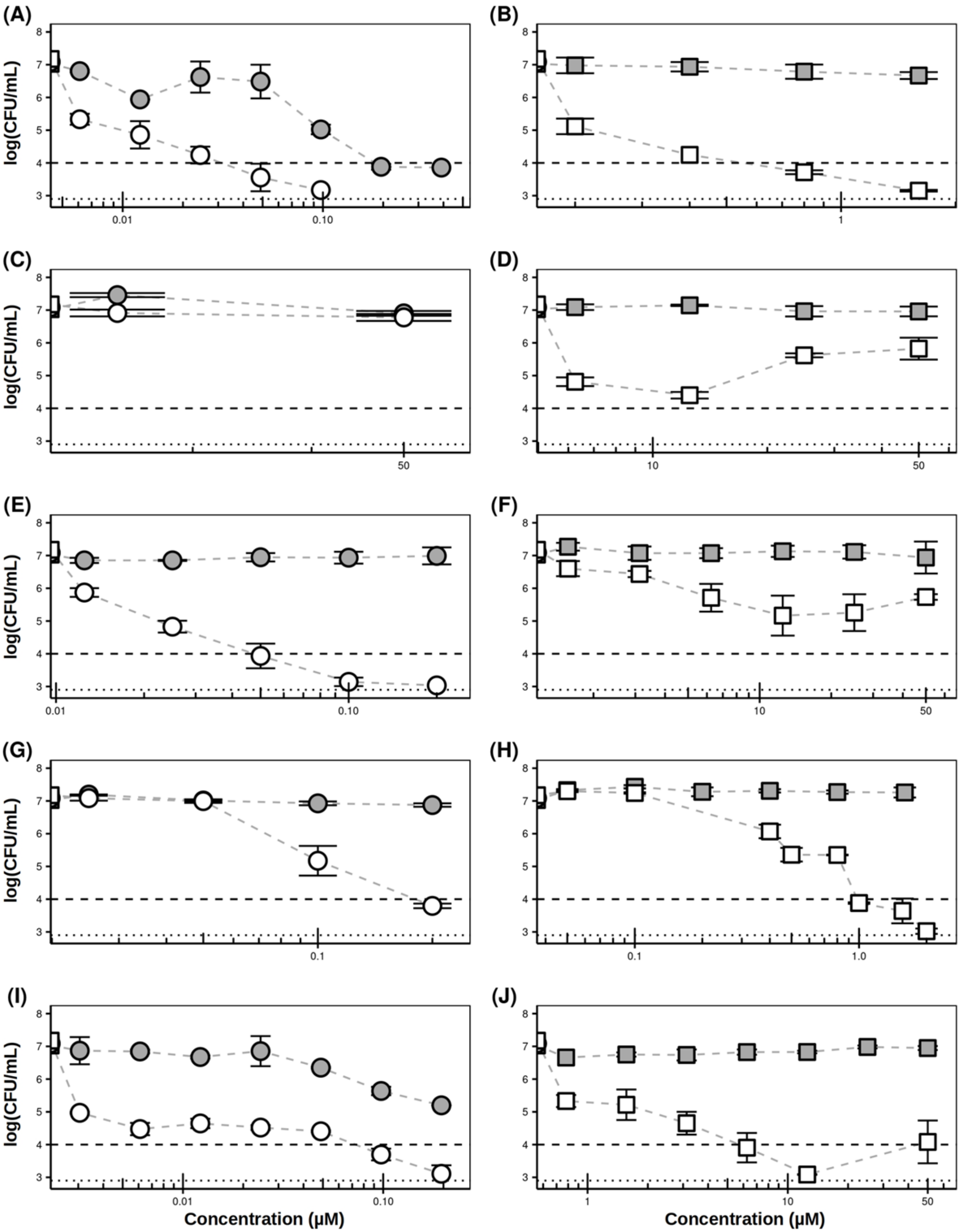

Supplementary Figure S12. Bacterial survival of *S. aureus*, after treatment with (A) **THPP**, (B) **THPP@AcLi**, (C) **T(OAc)PP**, (D) **T(OAc)PP@AcLi**, (E) **T(MAP)PP**, (F) **T(MAP)PP@AcLi**, (G) **T(PrOH)PyP**, (H) **T(PrOH)PyP@AcLi**, (I) **ZnTHPP** and (J) **ZnTHPP@AcLi**. Bacteria were incubated for 30 minutes and then irradiated with blue-LED light (15 J/cm^2, white symbols) or incubated in the dark (grey symbols). Afterwards, bacteria were diluted and plated in petri dishes, which were incubated in the dark 16 hours, before counting colonies. The dotted line indicates the limit of quantification (2.9 log), while the dashed line indicates a diminish of at least 3 log.

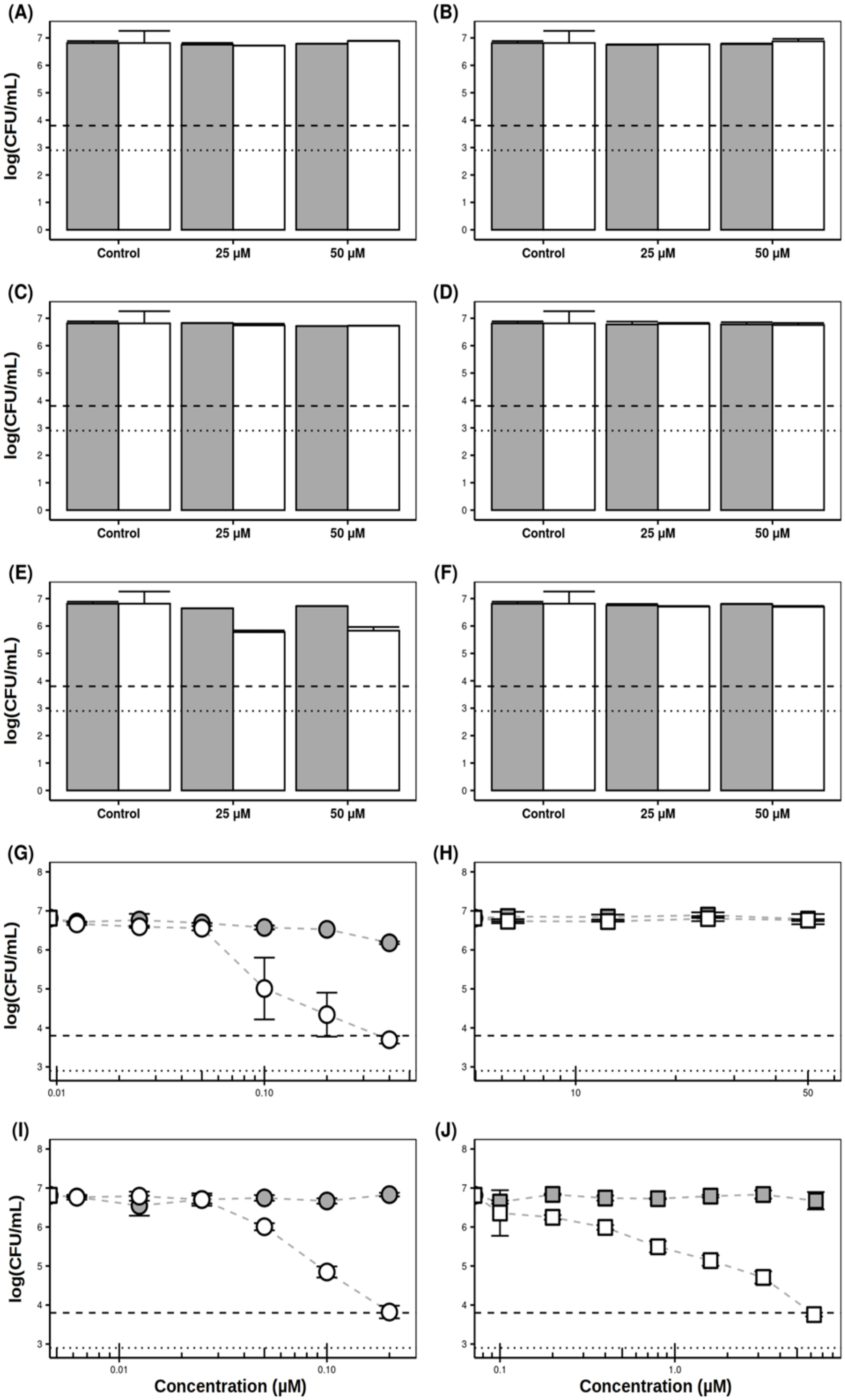

Supplementary Figure S13. Bacterial survival of *E. coli*, after treatment with (A) **THPP**, (B) **THPP@AcLi**, (C) **T(OAc)PP**, (D) **T(OAc)PP@AcLi**, (E) **T(MAP)PP**, (F) **T(MAP)PP@AcLi**, (G) **T(PrOH)PyP**, (H) **T(PrOH)PyP@AcLi**, (I) **ZnTHPP** and (J) **ZnTHPP@AcLi**. Bacteria were incubated for 30 minutes and then irradiated with blue-LED light (15 J/cm^2, white symbols) or incubated in the dark (grey symbols). Afterwards, bacteria were diluted and plated in petri dishes, which were incubated in the dark 16 hours, before counting colonies. The dotted line indicates the limit of quantification (2.9 log), while the dashed line indicates a diminish of at least 3 log.
